# The reality of virtual reality

**DOI:** 10.3389/fpsyg.2023.1093014

**Published:** 2023-02-15

**Authors:** Benjamin Schöne, Joanna Kisker, Leon Lange, Thomas Gruber, Sophia Sylvester, Roman Osinsky

**Affiliations:** ^1^Experimental Psychology I, Institute of Psychology, Osnabrück University, Osnabrück, Germany; ^2^Differential Psychology and Personality Research, Institute of Psychology, Osnabrück University, Osnabrück, Germany; ^3^Semantic Information Systems Research Group, Institute of Computer Science, Osnabrück University, Osnabrück, Germany

**Keywords:** virtual reality, EEG, HRV, anxiety, height

## Abstract

Virtual reality (VR) has become a popular tool for investigating human behavior and brain functions. Nevertheless, it is unclear whether VR constitutes an actual form of reality or is more like an advanced simulation. Determining the nature of VR has been mostly achieved by self-reported presence measurements, defined as the feeling of being submerged in the experience. However, subjective measurements might be prone to bias and, most importantly, do not allow for a comparison with real-life experiences. Here, we show that real-life and VR height exposures using 3D-360° videos are mostly indistinguishable on a psychophysiological level (EEG and HRV), while both differ from a conventional 2D laboratory setting. Using a fire truck, three groups of participants experienced a real-life (*N* = 25), a virtual (*N* = 24), or a 2D laboratory (*N* = 25) height exposure. Behavioral and psychophysiological results suggest that identical exogenous and endogenous cognitive as well as emotional mechanisms are deployed to process the real-life and virtual experience. Specifically, alpha- and theta-band oscillations in line with heart rate variability, indexing vigilance, and anxiety were barely indistinguishable between those two conditions, while they differed significantly from the laboratory setup. Sensory processing, as reflected by beta-band oscillations, exhibits a different pattern for all conditions, indicating further room for improving VR on a haptic level. In conclusion, the study shows that contemporary photorealistic VR setups are technologically capable of mimicking reality, thus paving the way for the investigation of real-world cognitive and emotional processes under controlled laboratory conditions. For a video summary, see https://youtu.be/fPIrIajpfiA.

## 1. Introduction

Getting into touch with virtual reality (VR), there is one fundamental question that arises immediately: How real is VR? The answer to that question will decide whether VR will only be considered an advanced form of computer technology, a next-generation PC, or if there is a categorical difference between VR and conventional (immersive) media experiences. The answer to this question is crucial for the application of VR for scientific purposes as a tool for learning and for therapeutic purposes.

However, at first glance, the question itself, whether VR is real, seems redundant, as the technology is labeled being a type of “reality” as most users describe an immersive experience. Surprisingly, there is little objective scientific evidence to support these introspective reports and there is little reason to believe that mounting a sophisticated monitor to the forehead indeed leads to an experience that can be considered a form of reality. By contrast, 3D cinema is not considered to create a form of reality albeit highly immersive. Whether VR actually can be considered “being real” is particularly difficult to answer as there is no technique or experiment to directly measure its reality or to derive its ability to create a feeling of reality from its technical properties. It is not possible to accurately quantify the degree to which a virtual reality (VR) experience might be perceived as “real” by an individual as this is a subjective question that depends on the individual’s perceptions. While there are various methods and techniques that can be used to measure brain activity and behavior in relation to VR experiences, these do not directly provide insights into an individual’s subjective experience of reality *per se*. However, comparing neural patterns and behavior during VR experiences to those during real-life experiences can potentially provide insights into the nature of VR and how it is processed by the brain as opposed to real-life experiences.

Consequently, the benchmark for assessing VR simulation quality can only be the user’s phenomenal consciousness, i.e., the subjective experience of being aware of one’s thoughts, feelings, and perceptions. Specifically, the crucial point is whether the brain can create an alternative reality from the sensory input provided by the VR device so that the brain itself regards this reality as sufficiently real overruling and replacing the physical reality it is actually situated in. To put it differently, the question is whether the sensory input provided by a VR headset is sufficient to create a phenomenal consciousness that is similar to or even indistinguishable from one constructed from real-life sensory input. Contemporary approaches determining the validity of virtual experiences follow this line of thought. One key aspect of VR is the feeling of being physically present in a scene, called “presence” (Sheridan, 1992; Slater et al., 1994).

Defining presence as the sensation of “being there” is the most common understanding of the concept, while its definition is widely debated (Murphy and Skarbez, 2022). Finding a definitive definition for the concept in question is beyond the scope of this study. For more extensive elaborations on the topic, see the studies by Schubert et al. (2001), Slater (2009), Nilsson et al. (2016), Slater (2018), and Murphy and Skarbez (2022). Nonetheless, three central factors are identified as particularly important in defining the feeling of reality in the present study. First, the sensory feed provided by the VR hardware comprises enough modalities in an adequate quality to relocate the user to the VR and to shield them from the physical reality—a technical ability often referred to as “immersion” (Nilsson et al., 2016). Especially regarding the visual sensory stream, VR offers a surrounding, stereoscopic, and thus enveloping representation of space (Murphy and Skarbez, 2022). Second, to create a feeling of presence, the artificial virtual environment has to present itself as a coherent plausible world following similar constraints as the real physical world in terms of realism (Welch et al., 1996; Slater, 2009; Kothgassner and Felnhofer, 2020). Third, presence is closely tied to agency (Piccione et al., 2019). A minimum requirement is that real head movement changes the viewing perspective in the virtual environment, thus physically locating the user in VR. Especially the latter invites us to visually explore the virtual scene, triggers sensations of awe (Chirico et al., 2018), and become immersed in the environment’s atmosphere (Felnhofer et al., 2015). This self-reinforcing phenomenon allows the physical world fade away.

Presence is typically a self-reported judgment, surveyed after a VR session using standardized questionnaires (IJsselsteijn et al., 2000; Riva et al., 2003), and thus highly prone to individual differences that arise from experiences with, e.g., computer games in general. Although presence gives a hint about how realistic an environment might have felt to the user, the questionnaire construction does not conclude how real the experiences are compared to reality. Presence solely serves the purpose of comparing the qualities of different virtual environments to each other (Usoh et al., 2000), but not assessing the simulation’s reality degree.

Overcoming these limitations of subjective post-induction measurements, the behavior during the virtual experience provides valid insights into the simulation’s realism [Freeman et al., 2000; Kisker et al., 2021a, see also Stoffregen et al. (2003)]. Conscious, deliberate responses to a virtual environment [e.g., refusal to cross great heights, see Kisker et al. (2021a)] and the instinctive reaction and automatic reflexes corresponding to real-life behavior [e.g., evasive action to physical objects or adaptions of posture, see Freeman et al. (2000)] imply that the underlying psychological mechanisms in VR resemble those under real-life conditions. This holds not only true for observable behavior but also for sympathetic responses such as enhanced skin conductivity (Gromer et al., 2019), heart rate (Gorini et al., 2010; Chittaro et al., 2017; Peterson et al., 2018; Kisker et al., 2021a), or any other way intense emotional responses might manifest [e.g., Kisker et al., 2021c, for review on emotion elicitation using VR, see Bernardo et al. (2021)]. Although behavior and psychophysical reactions observed under immersive VR conditions had been interpreted as resembling real behavior (Blascovich et al., 2002; Slater, 2009; Bohil et al., 2011; Lin, 2017; Kothgassner and Felnhofer, 2020; Kisker et al., 2021a), scientific certainty can only be reached when the behavioral and physiological reactions to a virtual scene are directly compared to those markers observed in the real environment that the scene mimics.

Determining VR’s degree of reality requires a triad. Not only the potential overlap between virtual and physical reality needs to be scientifically quantified, but also both need to be distinguished from conventional 2D computer simulations. Otherwise, it remains unclear which behavioral and physiological markers are hallmarks of realistic psychological functioning (see, e.g., Stoffregen et al., 2004). Only a few studies compare either VR or conventional setups with physical reality or even compare all of them in a triad. Furthermore, most studies that investigate VR in direct comparison to physical reality have a practical background and are based on clinical applications, foremost exposure therapies (Krijn et al., 2004; Gorini et al., 2010; Oing and Prescott, 2018; Boeldt et al., 2019). Although the use of VR as a therapeutic tool provides strong evidence that VR applications result in effective changes in real-life behavior and cognition even over the long term (Opriş et al., 2012), it needs to be considered that such findings are based on samples that tend to respond extremely to respective aversive stimuli (Cisler et al., 2010). Only recently, researchers venture into classifying VR with regard to its degree of reality based on norm samples. For example, emotional reactions to 360° footage of an enjoyable landscape resemble reactions to its real-life counterpart (Chirico and Gaggioli, 2019). Moreover, the perception of virtual height sufficiently evoked anxiety and reduced standing balance, not significantly differing from outcomes obtained from the equivalent real-life setup (Simeonov et al., 2005). However, to the best of our knowledge, no previous study has put an immersive 3D-360° VR setup into perspective by comparing it to both, an equivalent real-world setup and a conventional PC setup at the same time. Hence, the reality of VR is positioned in the theoretical “no-man’s land” which can be marked out in the border areas of physical reality and conventional computer simulations.

### 1.1. Current study and hypothesis

The study at hand aims to identify reliable electrophysiological, physiological, and subjective markers that distinguish between different degrees of reality in an environment that was experienced in physical reality, virtual reality by means of photorealistic 3D-360° videos, or conventional media. As a result, the study allows us to conclude to what degree virtual reality can be regarded as real. A height exposure paradigm was chosen to juxtapose these levels of reality for the following reasons: Reactions to virtual height exposure have been of interest from the very beginning of VR research and can to some extent be considered a classical application (Hodges et al., 1995; Rothbaum et al., 1995). Height exposure leverages the affordances of VR. The three-dimensionality instantly creates a strong sensation of height, triggering appropriate emotional and associated psychophysiological responses in most individuals. In other words, it is a proven, simple, almost classical way of eliciting stress responses in VR (Asjad et al., 2018; Gromer et al., 2018, 2019; Wolf et al., 2020; Kisker et al., 2021a). Previous attempts to directly compare VR and physical reality used rather calming nature scenes with mixed results. Although some studies concluded that VR elicits the same emotions as their real counterparts (Higuera-Trujillo et al., 2017; Chirico and Gaggioli, 2019), the latest meta-analysis states that reality surpasses virtual reality at least for positive emotions (Browning et al., 2020).

**FIGURE 1:**
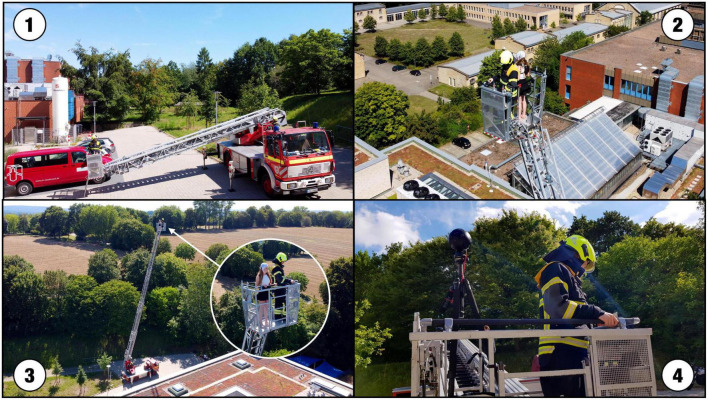
Photographs from the real-life setting: **(1)** Basket on the ground at the starting point. **(2)** Basket with firefighter and participant declining. **(3)** Basket at a maximum height of 33 m/108 feet. **(4)** Basket on the ground with firefighter and Insta 360 Pro VR camera instead of a participant. Photos taken by BS.

To maintain experimental control under realistic conditions as much as possible and to minimize motion artifacts that could significantly affect the quality of the EEG data, the method of choice was a fire truck bringing the participants up to a height of 33 m (Figure 1). The experimental conditions vary only slightly among the participants: Movements and the viewing gaze are restricted by the firefighter basket, a fixed time course consisting of an ascending phase, a maximum height, and a descending phase ensures high comparability of the experiences and thus a high data quality. To recreate this experience in VR and under conventional screen conditions, the ride in the firefighter basket was recorded several times with a VR video camera capable of stereoscopic 360° images (for details see Section “2. Materials and methods”). Stereoscopic 360° videos have previously been proven to be a viable option for creating believable virtual environments (Serino and Repetto, 2018) and were successfully utilized in scientific experiments (Higuera-Trujillo et al., 2017; Serino and Repetto, 2018; Schöne et al., 2019, 2020; Kisker et al., 2021b). Although the users’ location is fixed within the environment, it can be explored *via* head movements. Both, VR and conventional PC setups were identically designed except for the visual presentation form: In the VR version, the height exposure was mediated by a head-mounted display (HMD) depicting the fire basket video, whereas the very same video was mapped onto a 2D screen for the conventional PC experience. To render the virtual experience as authentic as possible with respect to the haptic and proprioceptive dimensions, a replica of the fire basket was built and complemented by unsteady ground and wind simulation. This mixed reality approach, aligning the visual impression of the height exposure with stereoscopic sound and haptics, is hypothesized to enhance immersion.

The rationale of this approach might at first glance seem at odds with the study’s aim to delimit VR settings from conventional screen experiences, but takes up a central thought about the nature of VR. In a devil’s advocate approach, all aspects of the experience which might factor into the sensation of reality were held constant—except the visual VR impression. The PC condition’s big screen occupying most of the participant’s field of view leads to low-level immersion [sometimes even referred to as desktop-VR, see Smith (2019)], and looking around the video *via* the keyboard, in turn, facilitates agency. Alternatively, the participants would sit in front of a much smaller monitor and passively watch the video. Yet, it would remain uncertain which of all the many differences between VR and 2D screen presentation would account for the differences in perception of reality. The proposed setup, however, allows us to pinpoint the difference to the HMD. On the other side, when no measurable difference between VR and 2D would arise, which given this devil’s advocate setup is much more likely, it would be hard to argue for a categorical difference between VR and conventional computer simulations with respect to their level of reality.

Following the general narrative of VR, we hypothesized that the difference in perceived realism should primarily manifest in a feeling of physical and emotional involvement, with reality being the most stressful experience as opposed to 2D being the least stressful one. VR was hypothesized to be somewhere between the two poles of this continuous scale of reality.

On a subjective level, self-reporting questionnaires should indicate the stronger presence and emotional involvement along this scale. Cardiovascular and electrophysiological measurements serve as objective markers. Heart rate variability (HRV) denotes the time variance between heartbeats (Shaffer and Ginsberg, 2017), and as it is controlled by the autonomous nervous system (ANS), it is a reliable indicator of resilience to stress and behavioral and emotional adaptability. Whereas a low HRV is interpreted as experiencing distress, a higher HRV indicates states of relaxation. The ANS modulates the intervals between two consecutive R-peaks of the QRS-complex (RR interval) through parasympathetic and sympathetic branches. Specifically, during mental stress, a shift in the autonomic balance of sympathetic activation and parasympathetic withdrawal occurs (Castaldo et al., 2015). The HRV is predominantly vagally controlled, however, on a physiological side, high-frequency oscillations reflect respiratory influences, low-frequency HRV indexes blood pressure control mechanisms, and very low-frequency HRV relates to kidney functions (Reyes del Paso et al., 2013).

Among other biomarkers such as hormone secretion, vasoconstriction of blood vessels, and increased blood pressure, the HRV reflects the “fight-or-flight” reaction (Taelman et al., 2008). High heart rate variability (HRV) is associated with resilience to stress and behavioral flexibility, while low HRV is associated with an increased stress response (Castaldo et al., 2015; An et al., 2020). Within the context of this paradigm, HRV should indicate if the level of stress elicited by the VR setting rather resembles the stress elicited by the real setting or by the feeling of safety of the 2D setting.

The power of cortical alpha- (8–12 Hz), theta- (4–7 Hz), and lower beta-band (15–20 Hz) oscillations were selected as electrophysiological markers for further investigation. Alpha and theta power are sensitive to stress, arousal, and anxiety (Schlink et al., 2017; Horvat et al., 2018; Klotzsche et al., 2018; Hofmann et al., 2019) with different functional properties. Alpha-band activity is associated with tonic alertness (Sadaghiani et al., 2010) and maintenance of attentional resources (Klimesch, 2012), it thus is believed to reflect unspecific alert and general vigilance (Davies and Krkovic, 1965) and correlates with anxiety (Knyazev et al., 2004). Overall, alpha functions are a promising marker for emotional arousal processing (Luft and Bhattacharya, 2015; Klotzsche et al., 2018). Whereas variations of alpha indicated exogenous orientated processing, i.e., threat-related external stimuli, theta serves as a marker for endogenous processes. Theta is mostly associated with cognitively demanding processes (Klimesch, 1999) and primarily encoding and related working memory processes (Jensen and Tesche, 2002; Sauseng et al., 2004). However, theta also varies as a function of emotion or affective involvement and indicates the cognitive processing of emotional stimuli. It thus might indicate emotion regulation (Ertl et al., 2013; Uusberg et al., 2014) or cognitive anxiety, i.e., perseverative worrying thoughts and feelings (Suetsugi et al., 2000; Gold et al., 2013; Moran et al., 2014; Putman et al., 2014; Lee et al., 2017). As an increase in theta has specifically been associated with the ability to reduce emotional reactions (Ertl et al., 2013), it might reflect active emotional regulation processing during the height exposure (see Grunwald et al., 2014). Whereas the alpha-band reflects the exogenous cortical response to the emotional arousal of the height exposure, the theta-band oscillations indicate the endogenous regulation of the experience. As mentioned earlier, VR has generally the ability to elicit strong emotions and especially reflex-like fear responses. Hence, it is even more important to assess whether the emotional reactions in VR, here negative arousal by means of height induction, are regulated like emotions elicited under real-life conditions or like emotions elicited by screen presentations.

Both, alpha- and theta-band oscillations have been proven effective for evaluating emotional arousal and stress in VR setups (Schlink et al., 2017; Klotzsche et al., 2018; Hofmann et al., 2019). Furthermore, beta-band oscillations have also been associated with numerous emotional processes (Bos, 2006; Schubring and Schupp, 2021). A considerably less recognized functional property that is more relevant for this study is that the beta-band also reflects somatosensory processing (Cheyne et al., 2003), sensory gating (Buchholz et al., 2014), motor planning, and execution (Campus et al., 2012). The beta-band even distinguishes between pleasant and unpleasant tactile stimulation on a single trial basis with high accuracy (Singh et al., 2014). Thus, it might effectively contribute to gaining insights into the quality and valence of the somatosensory information within this study’s context. In particular, it comes close to an objective marker of the otherwise subjective feeling of physical presence—one of the vital factors underlying VR.

In summary, our study aims at determining to what degree virtual reality simulates real-life experiences by comparing a virtual height exposure to corresponding real-life and a 2D monitor setup. Among other criteria, foremost the benchmark was whether the cognitive and emotional processes deployed by the brain to process the virtual experience resemble the ones in real-life or the monitor condition. Specifically, we investigated objective markers of emotional arousal, i.e., tonic alterness or vigilance, the regulation thereof, and the haptic processing by means of EEG and HRV, respectively.

## 2. Materials and methods

### 2.1. Participants

Seventy-nine participants were recruited from the Osnabrück University. The study was conducted in accordance with the Declaration of Helsinki and approved by the local ethics committee of Osnabrück University. Participants gave their informed written consent. They were screened for psychological and neurological disorders using a standard screening (anamnesis) and were excluded from participation if they suffered from a respective condition (e.g., affective disorders or epilepsy). All had a normal or corrected-to-normal vision. Five participants were excluded from the analysis due to insufficient data quality (*n* = 2) or technical problems during the ride in the fire truck’s basket (*n* = 3), resulting in a final sample size of *N* = 74 participants [Real-Life (RL) condition: *n* = 25, M_*age*_ = 25.32, SD_*age*_ = 4.28, 36.0% male, none diverse, 92.0% right-handed; Virtual Reality (VR) condition: *n* = 24, M_*age*_ = 22.29, SD_*age*_ = 2.82, 20.8% male, none diverse, 87.5.0% right-handed; and Computer (PC) condition: *n* = 25, M_*age*_ = 22.52, SD_*age*_ = 2.96, 12.0% male, none diverse, all right-handed]. Participants were rewarded with either partial course credits or 10€ for participation. As we recruited a random sample from the local student population, with most of them being psychology students, the sample is characterized by a high proportion of female students. It is well-known that gender imbalance might cause systematic biases (see, e.g., Perez, 2019). For example, women are more likely to suffer from anxiety disorders or general experiences of fear than men (e.g., McLean and Anderson, 2009). Some studies report that women are more prone to motion sickness (e.g., Chattha et al., 2020) and cybersickness (Shafer et al., 2017), while others do not find evidence for a gender bias in these factors [e.g., Wilson and Kinsela, 2017; Fulvio and Rokers, 2018; for review, see MacArthur et al. (2021)]. Yet, we assume that the gender imbalance did not affect the results obtained from group comparisons as we found no significant differences between groups before the height exposure, e.g., concerning trait anxiety, fear of height, avoidance of height, as well as positive and negative affects (see Section “3. Results”).

### 2.2. Conditions and setup

Participants were randomly assigned to one of three conditions: real-life (RL), virtual reality (VR), or computer screen (PC). The conditions were measured in a block-wise manner per condition as the measurements of the RL condition depended on the cooperating fire department’s schedule. However, the participants did not know in advance in which condition they would participate.

#### 2.2.1. Real-life condition (RL)

Participants were elevated in the firefighter’s basket of a fire truck up to 33 m height at a relatively remote and quiet part of the university’s campus (see Figure 1; Coordinates 52°16′57.5″N 8°01′12.9″E; viewing direction north). To ensure the participants’ safety, they were accompanied by a firefighter in the basket and the fire truck was operated by another firefighter from the ground. Both firefighters wore their full uniforms. The firefighter who accompanied the participants in the basket was instructed to turn his back on the participants throughout the ride and not to speak to them. Participants were instructed not to speak to the firefighter unless they wanted to quit the experiment early. The measurements of the RL condition were performed on 4 days in total.

#### 2.2.2. Virtual reality condition (VR)

The 3D-360° videos used for the VR condition were recorded with the Insta360Pro VR camera (Insta360, Shenzhen, China), at a frame rate of 60 fps, 4k resolution, and spatial sound. To cope with different weather conditions, wind force, changes in the environment, and time of day, the camera was set up on each day of the RL condition two to three times for recording. The elevation of the camera was performed exactly as with participants: the camera was accompanied by the same firefighter in full uniform, turning his back on the camera and not speaking at all. A total of 10 videos of the ride in the fire truck’s basket were recorded, two of which were not further used due to disturbed audio recording.

Participants in the VR condition were equipped with an HTC Vive Cosmos (HTC, Taoyuan, Taiwan) head-mounted display (HMD) which allows for a 3D-360° view and head tracking. The Cosmos HMD provides a resolution of 1440 × 1700 pixels per eye and spatial sound.

The videos were presented using the Simple VR video player (Simplevr.pro, Los Angeles, CA, USA). To enhance immersion and provide haptic feedback as similar as possible to the RL condition, participants were standing in a replica of the firefighter’s basket during the VR ride in the fire truck’s basket. The replica of the firefighter’s basket corresponded to the real basket in appearance and size and was positioned on unsteady ground. A fan was used to simulate wind.

#### 2.2.3. Computer condition (PC)

The very same video recordings as for the VR condition were used but presented in 2D instead of 3D-360°. The videos were presented in full-screen mode using GoPro VR Player (GoPro Inc, San Mateo, CA, USA). Participants were able to look around the video using the arrow keys on a conventional keyboard. Participants were positioned in front of an 86″ wide smartboard (SMART SBID-7086-v2; SMART Technologies ULC, Calgary, AB, Canada). The smartboard provided a resolution of 4k (3840 × 2160 pixels) and a frame rate of 60 fps. Equivalent to the VR condition, participants were standing in the same replica of the firefighter’s basket on unsteady ground, and a fan was used for wind simulation. The replica was positioned at a distance of 150 cm from the smartboard, resulting in a horizontal viewing angle of 2 × 32.33°.

### 2.3. Procedure

The experiment consisted of two appointments with a duration of approximately 75 min at the first and approximately 15 min at the second appointment. The ride in the fire truck’s basket was carried out during the first appointment. During the second appointment, the participants’ mood regarding the past ride in the fire truck’s basket was surveyed.

Before the ride in the fire truck’s basket (t0), participants filled in the German versions of the Positive and Negative Affect Schedule (PANAS, Krohne et al., 1996), the State-Trait-Anxiety-Inventory, trait version (STAI-T, Laux et al., 1981), the Acrophobia Questionnaire (AQ, Cohen, 1977), and the Sensation Seeking Scale Form-V (SSS-V, Zuckerman, 1996). The selection of the questionnaires was based on two previous studies investigating height in VR settings (Biedermann et al., 2017; Kisker et al., 2021a) and aimed to cover general affective responses, anxiety, and sensation seeking on the subjective level.

The mobile EEG and the ECG were applied by the test leaders (see Section “2.3.1. Electrophysiological recordings and preprocessing”). Afterward, the participants were led to the firetruck’s basket (real or replica). The participants saw neither the real basket nor the replica at any earlier time.

Participants were asked to stand still for 30 s facing the firetruck’s basket for ECG baseline measurement. The baseline of 30 s was chosen to determine the participants’ current state immediately before the ride in the basket. HRV is conventionally determined beginning at 5 min intervals, especially with respect to the diagnosis of cardiovascular disease (Salahuddin et al., 2007). Yet, previous studies showed that 30 s of measurement are sufficient to determine current mental stress using HRV in mobile setups [Cho et al., 2002; Salahuddin et al., 2007; for review, see Shaffer and Ginsberg (2017)]. Even much shorter baselines have been sufficient for EEG frequency analyses in conventional EEG (e.g., Gruber et al., 2008; Friese et al., 2013), mobile EEG (e.g., Packheiser et al., 2020, 2021; Lange and Osinsky, 2021), and combined VR-EEG studies (e.g., Kisker et al., 2021c; Schöne et al., 2021). After the baseline interval, participants entered the basket and were asked not to walk around in the basket during the ride. All participants were facing the same direction and were asked to lay their hands on the handrail. For the VR condition, participants were equipped with the Vive Cosmos HMD, which showed the still image of the starting position of the ride in the fire truck’s basket similar to the RL condition. For the PC condition, the same still image of the starting position was projected onto the smartboard. Afterward, the ascend of the basket started (real, VR, or video). When the highest point of 33 m was reached, the firefighter stopped the ascend and held the position for 63 s on average before descending again. The duration of the total ride and the timing of significant events (e.g., start of ascending, see Section “2.3.1. Electrophysiological recordings and preprocessing”) were individually recorded for each participant. The ride took 7.26 min on average (ascend: 2.6 min; descend: 3.7 min; see Section “3. Results”).

Directly after the ride in the fire truck’s basket (t1), participants filled in the German versions of the Igroup Presence Questionnaire (IPQ, Schubert et al., 2001) and the PANAS.

On the third day after the ride in the fire truck’s basket (t2, second appointment), the participants were asked to return to the laboratory to fill in the PANAS again and were debriefed.

#### 2.3.1. Electrophysiological recordings and preprocessing

For the EEG data acquisition, the mobile EEG system LiveAmp32 by Brain Products (Gilching, Germany) with active electrodes was used. The electrodes were applied in accordance with the international 10–20 system. An online reference (FCz) and ground electrode (AFz) were applied. A threshold of 25 kΩ is recommended for the used EEG system and the thresholds of 25, 20, and 15 kΩ have successfully been applied in previous studies using the same mobile EEG equipment (Burani et al., 2021; Lange and Osinsky, 2021; Liebherr et al., 2021; Visser et al., 2022). To ensure a good balance between signal quality and preparation time in our elaborate setting, the impedance of all electrodes was kept below 15 kΩ (see, e.g., Lange and Osinsky, 2021). The data were recorded with a sampling rate of 500 Hz and online band-pass filtered at 0.016–250 Hz. The following significant events of the ride in the fire truck’s basket were inserted as markers into the data based on the individual time measurement per participant: getting into the basket, starting the ascend, arriving at the highest point, starting the descend, and arriving at the starting position.

The EEG data were pre-processed using MATLAB (version R2020b, MathWorks Inc) and EEGLAB (Delorme and Makeig, 2004). The data were segmented into four epochs based on the individual time measurement, covering baseline, ascending phase, staying at the highest point, and descending phase. Per segment, each electrode was detrended separately. The continuous data were filtered between 1 and 30 Hz to reduce slow drifts and high-frequency artifacts. The electrodes were re-referenced to the average reference for further off-line analysis as recommended for large electrode sets (see, e.g., Cohen, 2014).

Artifact correction was performed per epoch by means of the “Fully Automated Statistical Thresholding for EEG artifact Reduction” (FASTER, Nolan et al., 2010). In brief, this procedure automatically detects and removes artifacts, such as blinks and white noise, based on statistical estimates for various aspects of the data, e.g., channel variance. FASTER has a high sensitivity and specificity for the detection of various artifacts and is described in more detail elsewhere (Nolan et al., 2010). Due to the recommendations for the use of FASTER with 32 channels, independent component analysis (ICA) and channel interpolation were applied, whereas channel rejection and epoch interpolation were not applied.

To isolate the theta-band (4–7 Hz), alpha-band (8–13 Hz), and lower beta-band (15–20 Hz) specific activity, each band power was calculated using a windowed fast Fourier transform (FFT). A hamming window with a length of 1 s and 50% overlap of the windows was applied. The mean FFT scores were calculated per electrode, logarithmized, and squared to determine the respective band power [ln(μV^2^)].

#### 2.3.2. Cardiovascular measurements and preprocessing

A three-channel ECG was applied and transmitted to the mobile EEG system *via* a BIP2AUX adapter by Brain Products (Gilching, Germany). The electrodes were placed at the left collarbone, the right collarbone, and the lowest left costal arch. The ECG was recorded synchronously with the EEG data.

The ECG data were segmented into epochs corresponding to the EEG epochs using MATLAB and further preprocessed using Brain Vision Analyzer. They were filtered between 5 and 45 Hz. A notch filter (50 Hz) was applied. An automatic R-peak detection was applied and counterchecked per visual inspection. The classical HRV parameters standard deviation of RR intervals (SDRR) and root mean square of successive differences (rmSSD) were calculated per phase (baseline, ascend, highest point, and descend) using MATLAB. The HRV parameters differed significantly between groups during baseline (see Section “3. Results”). To cope with these baseline differences, the individual changes in SDRR and rmSSD compared to the baseline were calculated for further analysis (delta = phase value − baseline value).

### 2.4. Statistical analysis

The statistical analyses were carried out using SPSS version 26. All variables were tested for normal distribution regarding the separate groups using the Shapiro–Wilk test. All further statistical tests were chosen accordingly (see Supplementary Table 6 for a detailed report of the Shapiro–Wilk test).

#### 2.4.1. Subjective measures

The scales of the questionnaires were calculated as sum scales. Concerning the PANAS, the scores for positive and negative affects concerning the time points before the ride in the fire truck’s basket (t0), directly after the ride (t1), and 3 days after (t2) were calculated. In addition, the change in affect was calculated as the difference between the pre-measurement and both post-measurements (change t1 = t1 − t0; change t2 = t2 − t0). PANAS, STAI, AQ, and IPQ were analyzed using the Kruskal–Wallis test and complemented by *post hoc* Mann–Whitney *U*-tests. The SSS-V was analyzed using a one-way ANOVA, complemented by *post hoc t*-tests.

#### 2.4.2. Dependent measures

Duration of the ride: To ensure that all participants experienced the ride in the fire truck’s basket for the same duration, the timing of the ride was compared using the Kruskal–Wallis test separately per phase as well as cumulated for the total ride.

Electrophysiological and cardiovascular measures: The EEG data and the HRV parameters were analyzed using the Kruskal–Wallis test and complemented by the *post hoc* Mann–Whitney *U*-tests. The HRV parameters were analyzed with respect to baseline and three separate phases (ascend, highest point, and descend) for an in-depth analysis. For the EEG data, the topographies were visually inspected and the electrodes of interest were determined using a false discovery rate (FDR, Benjamini and Yekutieli, 2001) based approach: the Kruskal–Wallis test and the *post hoc* Mann–Whitney *U*-tests were carried out per electrode, frequency band, and phase of the ride. The individual critical *p*-value indicating significant differences between groups was determined for each electrode. For further analysis, a whole brain analysis was performed, i.e., all electrodes were included in the mean values for statistical analysis as the FDR approach indicated significant differences between groups at the vast majority of electrodes. We successfully implemented a comparable analysis protocol for combined VR/EEG analysis yielding robust and meaningful data in previous studies (Kisker et al., 2021c; Schöne et al., 2021).

To provide statistical evidence for the equality of measures, we used the Wilcoxon two one-sided test (TOST) (1) as a robust equivalence test for all non-significant comparisons (Caldwell, 2022). The TOST procedure allows us to provide support for the null hypothesis, i.e., the absence of effects large enough to be considered worthwhile. In the TOST procedure, the smallest effect size of interest (SESOI) is determined to set upper and lower equivalence bounds. Two composite null hypotheses are tested: first, the effect found must be equal to or greater than the lower bound, and second, it must be equal to or smaller than the upper bound. If both can be statistically rejected, it can be concluded that the observed effects are practically equivalent to zero (Lakens, 2017).

**FIGURE 2:**
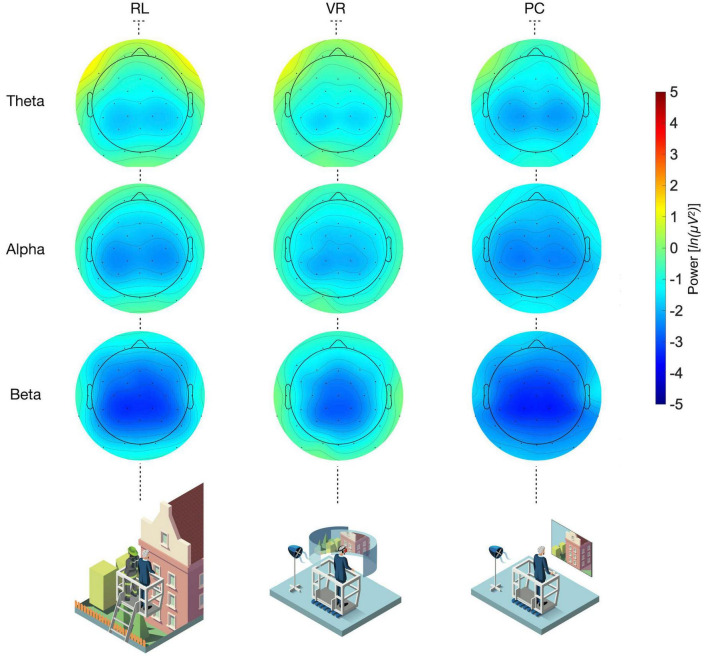
Average topographic oscillatory power amplitude [ln(μV2)] distribution across participants by frequency band (rows) and conditions (columns) while staying at the highest point of the ride in the fire trucks basket. The pictograms in the last row depict the experimental setup per condition. See Supplementary Figure 11 for the topographical distribution separately per phase and frequency band power.

We calculated the largest difference between groups in the baseline to determine the smallest SESOI for the comparisons in the alpha, theta, and beta bands. The rationale behind this approach is that for a difference between groups to be meaningful, i.e., being based on more than the simple baseline differences between the conditions, it has to surpass the largest differences present in the baseline. Hence, for alpha, we used 0.5199 ln(μV^2^), for theta 0.7455 ln(μV^2^), and for beta 0.4555 ln(μV^2^) (see also Figure 2).

## 3. Results

### 3.1. Duration of the ride

The duration of the separate phases as well as of the total ride did not differ between groups. The ascend took 2.5 min on average [*H*(2) = 2.76, *p* = 0.25; M_*RL*_ = 2.49, SD_*RL*_ = 0.30; M_*VR*_ = 2.54, SD_*VR*_ = 0.19; M_*PC*_ = 2.53, SD_*PC*_ = 0.19], and all participants stayed for 63 s at the highest point [*H*(2) = 0.6, *p* = 0.97; M_*RL*_ = 1.06, SD_*RL*_ = 0.05; M_*VR*_ = 1.06, SD_*VR*_ = 0.04; M_*PC*_ = 1.06, SD_*PC*_ = 0.04] and descended for 3.7 min on average [*H*(2) = 0.886, *p* = 0.65; M_*RL*_ = 3.75, SD_*RL*_ = 0.30; M_*VR*_ = 3.67, SD_*VR*_ = 0.33; M_*PC*_: 3.66, SD_*PC*_ = 0.36]. The total ride took an average of 7.26 min [*H*(2) = 0.24, *p* = 0.89; M_*RL*_ = 7.27, SD_*RL*_ = 0.55; M_*VR*_ = 7.27, SD_*VR*_ = 0.22; M_*PC*_: 7.25, SD_*PC*_ = 0.29].

### 3.2. Subjective measures

All groups were equal with respect to trait anxiety, fear of height, avoidance of height, as well as positive and negative affects before the ride in the fire truck’s basket [all *H*s(2) < 20, all *p*s > 0.05; see Supplementary Table 2], and sensation seeking [*F*_(2, 68)_ = 0.25, *p* = 0.78]. Furthermore, participants did not differ with respect to negative affect, neither directly after nor 3 days after the ride in the fire truck’s basket [all *H*s(2) < 50, all *p*s > 0.05; see Supplementary Table 2].

However, participants reported different sensations of presence, positive affect, and change in positive affect directly after the ride in the fire truck’s basket as well as 3 days later as a function of the experimental condition [all *H*s(2) > 24.00, all *p*s < 0.05, see Supplementary Table 2].

In particular, the RL group reported higher levels of general presence, spatial presence, and realness compared to both, VR and PC groups (all *U*s ≤ 151.0, all *p*s ≤ 0.003). Counterintuitively, the RL group reported lower levels of involvement compared to the VR group (*U* = 32.5, *p* < 0.001) and did not differ significantly from the PC group (*U* = 241.5, *p* = 0.64). Notably, the IPQ scale involvement compares the experienced environment with the real-life environment, which might be very ambiguous for the RL group to answer. The VR group reported significantly higher levels of presence regarding all IPQ scales compared to the PC group (all *U*s > 92.00, all *p*s ≤ 0.003, see Supplementary Table 3).

Moreover, the RL group experienced a stronger positive affect and reported higher changes in positive affect at t1 and t2 compared to both other groups (all *U*s ≤ 45.00, all *p*s < 0.05, see Supplementary Table 3). Furthermore, the VR group exhibited higher levels of positive affect and change in positive affect compared to the PC group (all *U*s ≤ 152.0, all *p*s < 0.03), with exception of the change in positive affect at t1 (*U* = 191.5, *p* = 0.074). See Supplementary Tables 2, 3, 8 for a detailed report of all statistics.

### 3.3. Alpha, theta, and beta powers

Overall, the Kruskal–Wallis test indicated significant differences between groups regarding each phase and each aforementioned frequency range [all *H*s(2) > 6.00; *p*s < 0.05; see Supplementary Table 9].

The RL group and the VR group exhibited similar levels of alpha and theta powers during all phases of the ride in the firetruck’s basket, significantly differing in beta power and during baseline in theta power only (all *p*s > 0.09, see Supplementary Table 4). In contrast, the PC group differed significantly from the RL group for each frequency band and each phase of the ride (all *p*s < 0.045, see Supplementary Table 4). In line, differences between VR and PC conditions were found for all phases and frequency bands as well, with exception of the baseline phases (see Supplementary Table 4).

**FIGURE 3:**
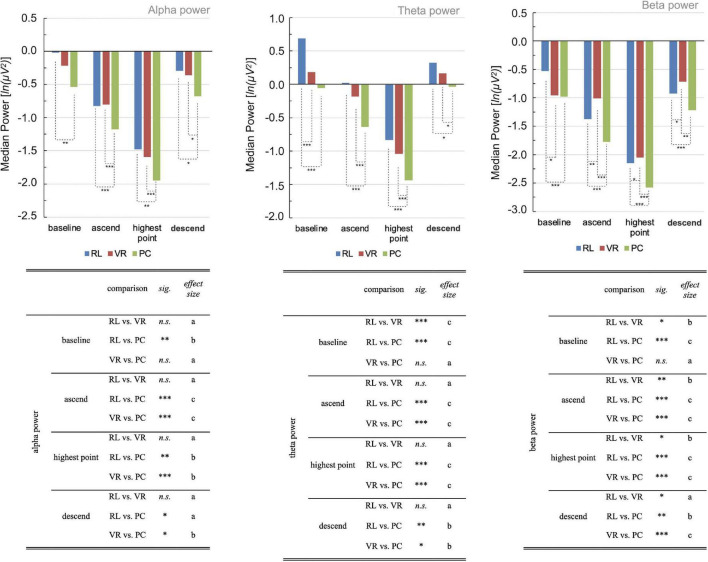
Median band power [ln(μV2)] per group, phase, and frequency band. Negative power values result from the logarithmic transformation during preprocessing: The logarithm of values greater than zero and smaller than one is negative. Hence, negative power values are to be read as smaller power compared to positive power values. The respective tables indicate the statistical characteristics per comparison in a reduced overview. Significant differences between groups are marked respectively (*p < 0.05, **p < 0.01; ***p ≤ 0.001), and the effect size r is interpreted as follows: a, small effect; b, medium effect; c, large effect. Find a detailed report of all statistics in the Supplementary material.

In more detail, the RL group exhibited considerably greater alpha- and theta-band powers compared to the PC group regarding all phases, but only slightly and non-significantly different levels compared to the VR group (all *p*s < 0.025, see Figures 2, 3; Supplementary Table 4). Diverging from the trend, beta power was highest for the VR group, lower for the RL group, and lowest for the PC group (see Figures 2, 3; Supplementary Table 4). Overall, the results suggest that the observed alpha and theta band effects in the RL and VR groups are largely similar, as indicated by the TOST procedure (Table 1), while both groups differ significantly from the PC condition (Figure 2).

**TABLE 1:** Wilcoxon TOST results for non-significant comparisons.

			TOST		
			***W***	***P***	**Effect size**
Alpha	Base	RLVR	207	0.032	0.277
		VRPC	209	0.035	0.237
	Ascend	RLVR	84	<0.001	-0.023
	High	RLVR	125	<0.001	0.027
	Descend	RLPC	219	0.036	0.331
		VRPC	190	0.014	0.38
Theta	Base	VRPC	109	<0.001	0.297
	Ascend	RLVR	50	<0.001	0.067
	High	RLVR	75	<0.001	0.167
	Descend	RLVR	100	<0.001	0.27
Beta	Base	VRPC	182	0.009	0.08

Wilcoxon TOST (W) of the upper equivalence bound and rank-biserial correlations as effect size. In rank-biserial correlations, zero indicates no relationship between the variables, positive values indicate the dominance of the first sample with 1 meaning that all values of the first sample are larger than all values of the second sample, and negative values indicate the dominance of the second sample, with −1 being the total dominance. Except this table, all tables reporting detailed statistics can be found in the Supplementary material. They are sorted by their relevance for the comprehension of the results.

See Supplementary Tables 4, 8, 9 for a detailed report of all statistics.

### 3.4. HRV parameters

The groups exhibited significant differences in SDRR [*H*(2) = 18.64, *p* < 0.001] and rmSSD [*H*(2) = 17.17, *p* < 0.001] during baseline. The baseline was recorded while participants stood in front of either the real fire truck’s basket (RL condition) or its replica (VR and PC conditions). Hence, instead of the uncorrected SDRR and rmSSD values, the changes in SDRR and rmSSD compared to the baseline (see Section “2. Materials and methods”) were further analyzed.

**FIGURE 4:**
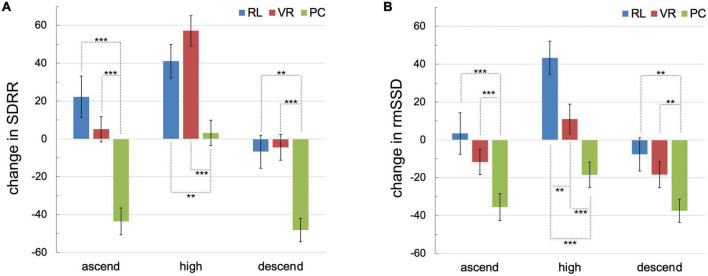
Changes in the HRV parameters SDRR **(A)** and rmSSD **(B)** for the separate phases of the ride in the fire truck’s basket compared to baseline per condition: The phases were corrected for the baseline (delta = HRV during the respective phase minus HRV during the baseline). The zero line thus marks the baseline, which was therefore not depicted separately. The error bars depict the standard deviation. Significant differences between groups are marked respectively (**p < 0.01, ***p < 0.001).

For an in-depth analysis, the HRV parameters were calculated and analyzed for the separate phases and corrected for baseline differences (delta = HRV during respective phase minus HRV during baseline; see also Figure 4). The Kruskal–Wallis test revealed significant differences in changes of SDRR and rmSSD as a function of the group regarding all separate phases [all *H*s(2) > 14.00, all *p*s ≤ 0.001; see Supplementary Table 10]. Specifically, RL and VR conditions did not differ for any phase and HRV parameter, with exception of the change in rmSDD during the phase at which participants stayed at the highest point of the ride. However, the PC condition significantly differed from both other conditions during all phases for both HRV parameters (see Figure 4; Supplementary Table 5). Notably, SDRR and rmSSD decreased during all phases compared to baseline regarding the PC condition and increased only slightly during the stay at the highest point (SDRR only, see Figure 4). On the contrary, the SDRR and rmSSD increased during the ascend and the stay at the highest point regarding the RL condition and deceased during the descend. The VR condition differed from this trend regarding the rmSSD during the stay at the highest point only (see Figure 4; Supplementary Table 5). See Supplementary Tables 5, 10 for a detailed report of all statistics.

## 4. Discussion

The study aimed to determine to what degree emotional and cognitive processing in virtual reality resembles the corresponding mechanisms deployed in a real-life setting as opposed to conventional laboratory conditions using objective markers. In turn, this allows estimating to which degree VR provides adequate sensory input for the brain from which it can construct a form of reality that it itself regards to be sufficient.

To this end, we set up a height exposure paradigm in which we shifted the degree of reality from a 2D monitor presentation to a VR simulation to a real experience in a parametric fashion. The premise by which it was assessed whether the VR experience can be considered sufficiently real to mimic a real-life experience was the simulation’s emotional potency. The real and the 2D monitor experience serve as the two opposite poles of a reality scale with VR located somewhere in between. The participants’ emotional experiences were indexed by several subjective measurements and further complemented by an objective measure of the ANS response (HRV), as well as electrophysiological correlates of arousal (alpha-band), emotion regulation (theta-band), and somatosensory processes (beta-band). The results shed new light on virtual experiences and confirm the introspective (or anecdotical) reports of VR users and scientific studies presuming that VR elicits real emotions while leaving a margin for further technological improvements on the somatosensory level.

Statistically speaking, the real-life condition and the virtual condition exhibit numerous non-significant differences across questionnaires and psychophysiological measurements, while they both differ from the PC condition. The overall pattern supports the notion that participants in real-life and virtual reality conditions generally exhibit the same level of alpha and theta powers (see below). Thus, when discussing the outcome of the study, the overall pattern of results is taken into consideration and not a single result is either significant or non-significant. Splitting the data into several phases makes the data more difficult to interpret and leads to a loss of statistical power, however, investigating the dynamic unfolding of cognitive and emotional processes with respect to the phases of the experiment (baseline, ascend, peak, and descend) seemed preferable to us from a scientific perspective. The baseline measurements foremost yield the weakest effects between VR and RL implying that with the increasing arousal from the height exposure, the differences between VR and RL seem to diminish. Therefore, the degree of reality that VR can evoke is closely related to its ability to evoke situational emotional responses.

### 4.1. Presence and interaction fidelity

In line with previous research, the VR condition generally elicited a stronger feeling of presence as opposed to the monitor condition. While immersive media like VR primarily might invoke presence using the perception of an enveloping space, they yet invoke realistic, most likely bottom-up mediated, responses (Murphy and Skarbez, 2022). A higher sense of presence is a crucial hallmark of VR especially in this experiment as it facilitates real-life behavior (Blascovich et al., 2002; Slater, 2009; Kisker et al., 2021a) and is in turn positively correlated with the emergence and strength of emotions, although the causal dynamics remain unspecified (Riva et al., 2007; Diemer et al., 2015; Felnhofer et al., 2015). A higher sense of presence in the VR condition as opposed to the monitor condition is thus a first indicator as well as a necessary precondition or accompaniment for real-life processing of VR content.

For the sake of completeness, we also obtained data for the real-life condition, showing that presence is highest for this condition. However, we suggest discarding this data as uninterpretable as the questionnaire is not meant for real-life application, and some questions cannot be answered meaningfully (Usoh et al., 2000). According to the data, a measurement artifact reflects this assumption as the self-reported involvement between the real and the monitor condition does not differ. We consider it imperative and essential to tackle this problem in the medium term. Immersion and presence are vital features of VR and thus need to be adequately measured to compare the score across different types of (simulated) reality. Notwithstanding the difficulties, the conventional questionnaire approach seems to fall short, especially as it poses a post-induction reappraisal method (IJsselsteijn et al., 2000; Usoh et al., 2000; Riva et al., 2003) and should be complemented, e.g., by objective and behavioral measures to estimate the fidelity of the simulation [e.g., Kisker et al., 2021a, see also Stoffregen et al. (2003)].

Since our setting was only limitedly interactive, it restricted the possibility to express behaviors. However, this restriction especially of body movements was desired to interfere as little as possible with the EEG measurement. Yet, the participants were not completely passive in this setting: They were able to control their field of view either by head-motion (RL and VR) which offered high interaction fidelity between both conditions (Shafer, 2021; see also, e.g., Riva, 2006; Cipresso et al., 2018; Kisker et al., 2021c) or were mediated by keystrokes (PC). Moreover, they were surrounded by a photorealistic 3D environment in both former conditions, while the PC condition was limited to a large 2D screen. Considering that, involvement should definitely differ between the former two and the latter conditions as high interaction fidelity increases the presence, interactivity, and realism (Shafer, 2021).

As Stoffregen et al. (2004) pointed out, direct comparisons of real-life and simulated environments are needed to investigate whether perception and (inter-)actions under both conditions are similar. However, these comparisons can only be realized to a limited extent based on subjective assessments. Moreover, presence is neither necessarily nor fully dependent on the knowledge that the experience is mediated by an immersive medium. For example, emotional experiences trigger coherent reactions despite the knowledge that the experience cannot be real (Kisker et al., 2021c) or the knowledge that one is wearing a VR headset (see Murphy and Skarbez, 2022). The feeling of presence is therefore not to be equated with the illusion of non-mediation (Murphy and Skarbez, 2022) and can be evoked *despite* the knowledge of mediation (Slater and Sanchez-Vives, 2016).

### 4.2. Emotions during and after the experiment

The comparison of the emotional responses between the three groups revealed an ambiguous pattern. Whereas positive affect differs substantially between all three conditions over time, negative affect did not differ at all. The ride thus was thrilling but perceived as a safe experience.

Interestingly, the pattern is reversed for the measurements obtained 3 days later. In retrospect, participants in the real and the VR condition rated their experience equally positive, whereas both groups differ from the PC condition. In contrast to the first pattern, this could indeed be interpreted as an indicator of VR being able to evoke real-life emotional responses. The PC condition, however, deviates. These contradicting results can be resolved by taking recruitment into account. To ensure that the collected data would come from a homogeneous sample, especially with respect to fear of heights, all participants were told that they would experience a real trip. Two groups of participants had to be disappointed, which might decrease the validity of the t1 data. The meaningfulness of the data regarding the perception of reality could be overshadowed by the disappointment of having missed a real ride.

Against the background of VR memory studies (Harman et al., 2017; Ernstsen et al., 2019; Krokos et al., 2019; Schöne et al., 2019; Smith, 2019; Kisker et al., 2020, 2021b), the comparison 3 days later is more informative. A common phenomenon comparing retrieval success for VR and conventional PC experiences is that immersive experiences are remembered with higher accuracy. Furthermore, behavioral (Schöne et al., 2019; Kisker et al., 2021b) and electrophysiological (Kisker et al., 2020) evidence suggest that different mnemonic processes are employed. Taken together, VR experiences seem to be weaved into a person’s narrative or autobiographical memory just like real experiences (Schöne et al., 2019; Kisker et al., 2020). Like episodic memory, the autobiographical memory is a subsystem of declarative memory (Renoult et al., 2012). Both are characterized by a unique first-person perspective and its encoding context. However, autobiographical memory comprises a larger set of operations, of which especially self-referential processing, personal relevance, and self-involvement are susceptible to virtual experiences (Greenberg and Rubin, 2003). Remembering VR experiences means remembering being at a place at a particular time and not only having it seen on a screen. Although the post-induction affects between the real and the VR condition differed (presumably due to disappointment), the event is emotionally remembered in the most similar way. This finding seems to be more conclusive within the context of VR applications than the immediate post-induction measurement: Although VR is perhaps less exciting than a real-life ride, it is remembered as exciting. The efficacy of, e.g., therapeutical and educational applications depends on this aspect of VR. Long-term success can only be achieved when the virtual application substitutes not only on a factual but also on an emotional level for the corresponding real-life scenario: Virtual treatment of acrophobia (Renoult et al., 2012), bulimia (De Carvalho et al., 2017), or social anxiety (Bouchard et al., 2017) is effective if patients remember the exposure emotionally as if it was a real experience.

### 4.3. Heart rate variability

Although emotional memory provides a good starting point for evaluating VR’s realness, online measures are needed to draw a clear picture of the participants’ mental stress during the ride. To assess the level of reality, VR, and PC simulations, the HRV functions as a marker for stress imposed by the conditions on the autonomic nervous system (ANS, Shaffer and Ginsberg, 2017).

Two measures are used to evaluate the variance of the RR intervals; the standard deviation (SDRR) and the root mean square of successive RR interval differences (rmSSD). Both measures are commonly employed and appropriate ultra-short time measurements (<5 min) (Baek et al., 2015; Shaffer and Ginsberg, 2017). The basis for the short-term HRV is the aforementioned interplay between sympathetic and parasympathetic branches, but also respiration-driven speeding and slowing of the heart, baroreceptor reflex, and rhythmic changes in vascular tone [for a comprehensive overview, see Shaffer and Ginsberg (2017)]. SDRR and rmSSD reflect slightly different ANS properties, with the rmSSD reflecting the vagally mediated changes in variance (Shaffer and Ginsberg, 2017). Since there is a debate about whether one measurement should be favored over the other one predominantly arising from a lack of research about their physiological origin (Shaffer and Ginsberg, 2017), both measurements were used in this study to estimate the ANS stress response.

The reported data are baseline corrected; however, another baseline was chosen as opposed to the questionnaires to circumvent the anticipated problem of disappointment. To assess the ride’s emotional efficacy, the baseline was measured while standing in front of the fire truck basket or the replica, making it clear to the participants what they had to expect. While this approach avoids the problems already mentioned, on the downside, it does not provide an entirely emotionally neutral baseline. The participants would joyfully expect a real ride or be aware that they would participate in an unconventional but neutral experiment. The very nature of this unconventional experiment makes it impossible to find the ideal baseline.

As higher HRV scores indicate relaxation and lower scores indicate stress, a baseline-corrected negative score indicates an increase in stress and a positive score indicates relaxation. The responses in all phases are identical for the SDRR showing that the stress response did not differ between the real and the VR condition. The rmSSD followed the same trend, except for the response at the highest point. Both groups differed significantly from the PC condition in all phases and both measures: The baseline-corrected data indicate an elevated stress response in the PC condition and a likewise light to neutral stress response in the real and virtual conditions.

This overall pattern unfolds the temporal dynamic of the emotional experiences. The significance pattern for the SDRR phase analysis confirms the first impression that the real and virtual conditions are indistinguishable in terms of ANS responses. Positive scores for both conditions in the ascend even increasing in the peak phase, corresponding to the overall positive PANAS ratings, indicate a vagotonic state of the ANS, associated with relaxation or a positive mood (Shiota et al., 2011). During the descent, the score again leans toward the baseline. The PC condition follows a similar course; however, the peak resembles the baseline indicating an overall dissatisfaction and an ergotropic state of the ANS (Gellhorn, 1970). The rmSSD pattern follows the course with a deviation at the peak: Reality surpasses VR significantly. Since the significance patterns otherwise support the equivalence of reality and VR, the difference might be explained physiologically. Both measurements reflect slightly different ANS properties with the rmSSD reflecting the vagally mediated respiration-driven speeding and slowing of the heart (Schipke, 1999). Hence, it might be a different respiratory pattern that accounts for the differences. Being outside, experiencing a fresh breeze at 33 m, when the temperature at the ground was beyond 30°C, and feeling a sensation of awe (Chirico et al., 2017) could trigger a deep breath and thus increase the score technically.

As an interim conclusion, the HRV data corresponds to self-reported emotional memory, revealing the same equivalence pattern of the real and virtual domains in distinction to the PC condition at t2. They thus confirm a previous VR video study providing the first evidence that heart rate and by that the visceral ANS stress response to VR environments corresponds to real-life responses much more than to conventional laboratory settings (Higuera-Trujillo et al., 2017).

### 4.4. Electrophysiological correlates

While the HRV analysis provides robust evidence for a somatic level of arousal, the EEG analysis is more conclusive over a wider variety of cognitive, emotional, and somatosensory processes. Interestingly, the overall picture indicates that the EEG result parallels what a naive VR user would have suspected: The emotional responses elicited by the real-life and the virtual experience are indistinguishable on an electrophysiological level as the alpha- and theta-band oscillations do not yield significant differences. However, the participants’ somatosensory experiences differed between all three conditions as indexed by beta-band oscillations.

First of all, it should be noted that we employed a robust methodological approach at the expense of the EEG’s already limited ability to identify the neural underpinning of the obtained signal. Thus, a distinct functional attribution of the respective oscillations remains speculative to a certain degree and leaves a margin for further improvements. Importantly, a more precise classification of the functional properties does not necessarily augment the epistemological value of this particular study. The brain’s oscillatory response to VR by means of 3D-360° videos resembles the response to a real-life event in two of the three selected frequency bands. Regarding alpha and theta band powers, the equivalence tests (TOST) strongly indicate that the effects for RL and VR are equivalent. On the other side, differences between RL and PC as well as VR and PC mainly exhibit strong effects.

Generally, as alpha-band oscillations are correlating with emotional arousal (Klotzsche et al., 2018; Schubring et al., 2020), the data indicate that VR comes in with the same sense of arousal (or thrill) as the real-life ride. The advantage of the study’s experimental setup is that even the real-life situation with a professional firefighter crew imparted a feeling of safety and assurance. Aside from these safety considerations, it should be noted that the alpha-power decreased with gaining altitude till the highest point, and then got back closer to zero. As the alpha-band is inversely correlated with cortical activity (Schöne et al., 2016), both measures, HRV and alpha power, likewise imply heightened stress or arousal with a gaining altitude. In other words, the 3D/360° VR video and the real-life condition conveyed the same level of hazard and, as pointed out in the Introduction section, likewise facilitated the same level of tonic, unspecific alertness, and anxiety but also general vigilance or sustained attention (Dockree et al., 2007). Interestingly, theta oscillations are most positive in the baseline for the RL condition as opposed to VR and PC: An increase in spectral theta has been interpreted as reflecting an integrative regulatory function (Cruikshank et al., 2012). Whereas the positive theta oscillations indicate the downregulation of negative emotions, like it would be expected when standing in front of the firetruck ready for departure, the negative theta values reflect a diminished or even a loss of emotional control (Grunwald et al., 2014). This can be either a deliberate process, e.g., due to an overwhelming experience, or the lack of necessity. The latter seems to apply to the PC condition, where theta is most negative: Emotion regulation appears to be of minimal importance in this context, as the experience is only displayed on a screen. Conventional media elicits emotions that are less impactful and are accordingly managed differently. Emotions are a response to the environment in which they arise and prompt appropriate actions. This link between emotion and environment is dissolved or at least weakened when the current emotional state is triggered by a monitor experience. For example, anxiety elicited by a screening event does not provide information about the hazardousness of the environment, often it is even elicited for entertainment purposes by watching a horror movie. In a corresponding real-life situation, it facilitates appropriate behavior and has to be regulated properly. Processes that also occur in VR (Kisker et al., 2021a,c), which is why it is in line with previous literature to the study at hand, and real life do not exhibit significant differences with respect to the brain’s theta-band response. The emotions elicited by the height exposure need to be evaluated and regulated appropriately. This cascade makes a case for the realism of VR. VR not only seems to be sufficiently real to elicit genuine emotions (first response) but once provoked, the brain processes them like real emotions; and seems to deploy the same emotion regulation mechanisms as if they were evoked under real-life conditions (second response). Denying the authenticity of an experience or an emotion is a well-known way to regulate, e.g., fear (Cantor, 2006) and it would be a possible strategy in the VR condition by discarding VR as being unreal. A feeling is only denoted as imagination or fiction to alleviate it (e.g., cognitive avoidance, Lin, 2017). Given the premise that the obtained theta signal in our study possesses sufficient discriminatory power for regulatory processes, the data imply that this demotion to the imagination is not applied to our experiment.

As an intermediate conclusion, the PANAS questionnaire (at t2), the HRV measurements, and the alpha and theta oscillations indicate—individually and in conjunction—that emotions elicited in VR resemble real-life emotions. The study thus fills the gap in VR research showing that VR simulations elicit strong realistic emotions (Gorini et al., 2010; Felnhofer et al., 2015; Chirico and Gaggioli, 2019; Gromer et al., 2019; Bernardo et al., 2021) and supports the assumption that they reflect real-life emotional processing (Kisker et al., 2021a,c).

However, the beta-band results exhibit a different pattern: All three conditions differ from each other. As mentioned in the introduction, the beta-band has also been associated with emotional processes, but as all previously discussed metrics imply emotional equivalence between VR and real-life scenarios, the somatosensory account seems to be preferable. Technically speaking, the experiment was a mixed reality (MR) setup as the study aimed to minimize the perceptual differences between all three conditions by including physical cues, e.g., a wobbly basket replica and wind simulation. All three conditions still exhibited unique somatosensory characteristics: especially the HMD sticks out. Only the participants in the VR condition had additional weight mounted on the head, influencing proprioceptive perception. It is unclear to what extent VR (or MR) environments need to physically resemble a corresponding real-world scenario to mitigate such somatosensory differences. Lighter HMDs, synthetic skin mimicking touch sensation (Lucarotti et al., 2013), and other technical advances might further enhance the realism of simulations. Furthermore, the RL condition was the only one that actually came with a vertical acceleration. Nevertheless, according to the data, the study’s setup was sufficiently real to elicit real-life emotions, leaving the question of to which degree physical cues play a role at all. Notably, methodological examinations demonstrated that wearing common VR headsets like the HTC Vive does not impact the EEG’s signal quality regarding frequency bands below 50 Hz (Hertweck et al., 2019). In a competition for cognitive and especially attentional resources, sensory information might only play a subordinate role to emotional cues, especially in a scenario with a fixed body position and limited interaction. Conclusively, it seems that the relevance of somatosensory information for the manifestation of a sense of realism highly depends on the purpose of the application.

Alternatively, ongoing beta activity is associated with subsequent memory formation success, independent of stimulus modality (Scholz et al., 2017). As the ride was a unique experience, the beta power might also reflect a memory-promoting state that albeit being modality independent might be modulated by attentional or emotional states accounting for the differences between the conditions.

## 5. Conclusion

The study has shown that today’s VR setups using photorealistic 3D-360° experiences fulfill the essential prerequisites for the emergence of a feeling of reality and paves the way for a more in-depth examination of the relevant cognitive and emotional processes as well as the technological features of VR giving rise to them. Furthermore, the study provides a scientific framework for developing recreational, educational, and therapeutic VR applications. Scientists might proportionally benefit from the enhanced ecological validity achieved by VR. Psychological processes can be studied under previously unprecedented realistic conditions in controlled laboratory conditions (Bohil et al., 2011; Parsons, 2015). However, as outlined in the introduction, the realism of VR simulations requires particular diligence in applying VR materials. The laws and guidelines, according to which content is created and evaluated for an audience (e.g., the MPA rating system in the USA, scientific ethics committees), do not translate into the virtual domain without any adjustment (Slater et al., 2020) as VR can be considered to be sufficiently real to mimic reality. For a video summary, see https://youtu.be/fPIrIajpfiA.

## 6. Limitations

For practical reasons, our study investigated only one scenario under real-life, VR, and monitor conditions. It is yet unclear to which condition or other scenarios our results generalize. As the Introduction section mentioned, height exposures are classic immersive VR experiences leveraging all its affordances. Future research should investigate less immersive and less arousing scenarios to determine a cutoff where VR and PC might be on the same level, both differing from reality. However, the conclusions drawn from our study are in line with previous VR studies from various fields inferring the degree of reality of virtual reality from behavioral observations (see Section “1. Introduction”). The generalizability of our research thus should be given and is presumably higher than the generalizability of monitor experiments.

The HRV baseline chosen for the experiment was not free of induction. Standing in front of the fire truck expecting to go up naturally led to increased arousal. The rationale behind this approach was to correct for any condition’s specific arousal by subtracting this baseline from the HRV during the ride. However, an induction-free baseline might shed a more differentiated light on the temporal dynamics of the arousal during the experience.

The participant in the experiment had no task but was passively exposed to the height. Thus, tying the electrophysiological responses to mental states is speculative beyond very basic functions. Nonetheless, due to the very simple measurements, future research should include behavior and investigate task-specific neural oscillation for that reason.

## Data availability statement

The datasets presented in this study can be found in online repositories. The names of the repository/repositories and accession number(s) can be found below: The data sets generated and analyzed during the current study are available in the Open Science Framework (OSF) repository: https://osf.io/cu2pz/?view_only=6763bdce7219449184a0c25ee8f95055.

## Ethics statement

The studies involving human participants were reviewed and approved by Local Ethics Committee of Osnabrück University. The participants provided their written informed consent to participate in this study.

## Author contributions

JK, SS, and LL performed the testing and data collection. JK, SS, and LL performed the data analyses under the supervision of BS. BS performed the data interpretation and drafted the manuscript. JK, LL, TG, and RO provided the critical revisions. BS, JK, LL, TG, SS, and RO contributed to the study design based on an idea by BS. All authors approved the final version of the manuscript for submission.

## References

[B1] An E. Nolty A. A. T. Amano S. S. Rizzo A. A. Buckwalter J. G. Rensberger J. (2020). Heart rate variability as an index of resilience. *Milit. Med.* 185 363–369. 10.1093/milmed/usz325 31642481

[B2] Asjad N. S. Paris R. Adams H. Bodenheimer B. (2018). “Perception of height in virtual reality – a study of climbing stairs,” in *Proceedings of the SAP 2018: ACM symposium on applied perception*, (Vancouver, BC), 1–8. 10.1145/3225153.3225171

[B3] Baek H. J. Cho C. H. Cho J. Woo J. M. (2015). Reliability of ultra-short-term analysis as a surrogate of standard 5-min analysis of heart rate variability. *Telemed. e-Health* 21 404–414. 10.1089/tmj.2014.0104 25807067

[B4] Benjamini Y. Yekutieli D. (2001). The control of the false discovery rate in multiple testing under dependency. *Ann. Stat.* 29 1165–1188. 10.1214/aos/1013699998

[B5] Bernardo P. D. Bains A. Westwood S. (2021). Mood induction using virtual reality: A systematic review of recent findings. *J. Technol. Behav. Sci.* 6, 3–24. 10.1007/s41347-020-00152-9

[B6] Biedermann S. V. Biedermann D. G. Wenzlaff F. Kurjak T. Nouri S. Auer M. K. (2017). An elevated plus-maze in mixed reality for studying human anxiety-related behavior. *BMC Biol.* 15:125. 10.1186/s12915-017-0463-6 29268740 PMC5740602

[B7] Blascovich J. Loomis J. Beall A. C. Swinth K. R. Hoyt C. L. Bailenson J. N. (2002). Immersive virtual environment technology as a methodological tool for social psychology. *Psychol. Inq.* 13 103–124. 10.1207/S15327965PLI1302_01

[B8] Boeldt D. McMahon E. McFaul M. Greenleaf W. (2019). Using virtual reality exposure therapy to enhance treatment of anxiety disorders: Identifying areas of clinical adoption and potential obstacles. *Front. Psychiatry* 10:773. 10.3389/fpsyt.2019.00773 31708821 PMC6823515

[B9] Bohil C. J. Alicea B. Biocca F. A. (2011). Virtual reality in neuroscience research and therapy. *Nat. Rev. Neurosci.* 12 752–762. 10.1038/nrn3122 22048061

[B10] Bos D. O. (2006). EEG-based emotion recognition. *Influence Vis. Auditory. Stimuli* 56, 1–17.

[B11] Bouchard S. Dumoulin S. Robillard G. Guitard T. Klinger E. Forget H. (2017). Virtual reality compared with in vivo exposure in the treatment of social anxiety disorder: A three-arm randomised controlled trial. *Br. J. Psychiatry* 210 276–283. 10.1192/bjp.bp.116.184234 27979818

[B12] Browning M. H. E. M. Shipley N. McAnirlin O. Becker D. Yu C.-P. Hartig T. (2020). An actual natural setting improves mood better than its virtual counterpart: A meta-analysis of experimental data. *Front. Psychol.* 11:2200. 10.3389/fpsyg.2020.02200 33101104 PMC7554239

[B13] Buchholz V. N. Jensen O. Medendorp W. P. (2014). Different roles of alpha and beta-band oscillations in anticipatory sensorimotor gating. *Front. Hum. Neurosci.* 8:446. 10.3389/fnhum.2014.00446 24987348 PMC4060639

[B14] Burani K. Gallyer A. Ryan J. Jordan C. Joiner T. Hajcak G. (2021). Acute stress reduces reward-related neural activity: Evidence from the reward positivity. *Stress* 24 833–839. 10.1080/10253890.2021.1929164 33998959

[B15] Caldwell A. R. (2022). Exploring equivalence testing with the updated TOSTER R package. *PsyArXiv* [Preprint]. 10.31234/osf.io/ty8de

[B16] Campus C. Brayda L. de Carli F. Chellali R. Famà F. Bruzzo C. (2012). Tactile exploration of virtual objects for blind and sighted people: The role of beta 1 EEG band in sensory substitution and supramodal mental mapping. *J. Neurophysiol.* 107 2713–2729. 10.1152/jn.00624.2011 22338024 PMC3362272

[B17] Cantor J. (2006). “Why horror doesn’t die: The enduring and paradoxical effects of frightening entertainment,” in *Psychology of entertainment*, eds Bryant J. Vorderer P. (Hillsdale, NJ: Lawrence Erlbaum Associates Publishers), 315–327.

[B18] Castaldo R. Melillo P. Bracale U. Caserta M. Triassi M. Pecchia L. (2015). Acute mental stress assessment via short term HRV analysis in healthy adults: A systematic review with meta-analysis. *Biomed. Sign. Proc. Cont.* 18 370–377. 10.1016/j.bspc.2015.02.012

[B19] Chattha U. A. Janjua U. I. Anwar F. Madni T. M. Cheema M. F. Janjua S. I. (2020). Motion sickness in virtual reality: An empirical evaluation. *IEEE Access* 8 130486–130499.

[B20] Cheyne D. Gaetz W. Garnero L. Lachaux J.-P. Ducorps A. Schwartz D. (2003). Neuromagnetic imaging of cortical oscillations accompanying tactile stimulation. *Cogn. Brain Res.* 17 599–611. 10.1016/S0926-6410(03)00173-3 14561448

[B21] Chirico A. Gaggioli A. (2019). When virtual feels real: Comparing emotional responses and presence in virtual and natural environments. *Cyberpsychol. Behav. Soc. Network.* 22 220–226. 10.1089/cyber.2018.0393 30730222

[B22] Chirico A. Cipresso P. Yaden D. B. Biassoni F. Riva G. Gaggioli A. (2017). Effectiveness of immersive videos in inducing awe: An experimental study. *Sci. Rep.* 7:1218. 10.1038/s41598-017-01242-0 28450730 PMC5430774

[B23] Chirico A. Ferrise F. Cordella L. Gaggioli A. (2018). Designing awe in virtual reality: An experimental study. *Front. Psychol.* 8:2351. 10.3389/fpsyg.2017.02351 29403409 PMC5786556

[B24] Chittaro L. Sioni R. Crescentini C. Fabbro F. (2017). Mortality salience in virtual reality experiences and its effects on users’ attitudes towards risk. *Int. J. Hum. Comput. Stud.* 101 10–22. 10.1016/j.ijhcs.2017.01.002

[B25] Cho B. H. Lee J.-M. Ku J. H. Jang D. P. Kim J. S. Kim I.-Y. (2002). “Attention enhancement system using virtual reality and EEG biofeedback,” in *Proceedings IEEE virtual reality 2002*, (Orlando, FL: IEEE), 156–163.

[B26] Cipresso P. Giglioli I. A. C. Raya M. A. Riva G. (2018). The past, present, and future of virtual and augmented reality research: A network and cluster analysis of the literature. *Front. Psychol.* 9:2086. 10.3389/fpsyg.2018.02086 30459681 PMC6232426

[B27] Cisler J. M. Olatunji B. O. Feldner M. T. Forsyth J. P. (2010). Emotion regulation and the anxiety disorders: An integrative review. *J. Psychopathol. Behav. Assess.* 32 68–82. 10.1007/s10862-009-9161-1 20622981 PMC2901125

[B28] Cohen D. C. (1977). Comparison of self-report and overt-behavioral procedures for assessing acrophobia. *Behav. Ther.* 8 17–23. 10.1016/S0005-7894(77)80116-0

[B29] Cohen M. X. (2014). *Analyzing neural time series data: Theory and practice.* Cambridge, MA: MIT press, 10.7551/mitpress/9609.001.0001

[B30] Cruikshank L. C. Singhal A. Hueppelsheuser M. Caplan J. B. (2012). Theta oscillations reflect a putative neural mechanism for human sensorimotor integration. *J. Neurophysiol.* 107 65–77. 10.1152/jn.00893.2010 21975453

[B31] Davies D. R. Krkovic A. (1965). Skin-conductance, alpha-acitivity, and vigilance. *Am. J. Psychol.* 78 304–306. 10.2307/1420507 14290763

[B32] De Carvalho M. R. De Santana Dias T. R. Duchesne M. Nardi A. E. Appolinario J. C. (2017). Virtual reality as a promising strategy in the assessment and treatment of bulimia nervosa and binge eating disorder: A systematic review. *Behav. Sci.* 7:43. 10.3390/bs7030043 28698483 PMC5618051

[B33] Delorme A. Makeig S. (2004). EEGLAB: An open source toolbox for analysis of single-trial EEG dynamics including independent component analysis. *J. Neurosci. Methods* 134 9–21. 10.1016/j.jneumeth.2003.10.009 15102499

[B34] Diemer J. Alpers G. W. Peperkorn H. M. Shiban Y. Mühlberger A. (2015). The impact of perception and presence on emotional reactions: A review of research in virtual reality. *Front. Psychol.* 6:26. 10.3389/fpsyg.2015.00026 25688218 PMC4311610

[B35] Dockree P. M. Kelly S. P. Foxe J. J. Reilly R. B. Robertson I. H. (2007). Optimal sustained attention is linked to the spectral content of background EEG activity: Greater ongoing tonic alpha (∼10 Hz) power supports successful phasic goal activation. *Eur. J. Neurosci.* 25 900–907. 10.1111/j.1460-9568.2007.05324.x 17328783

[B36] Ernstsen J. Mallam S. C. Nazir S. (2019). Incidental memory recall in virtual reality: An empirical investigation. *Proc. Hum. Fact. Ergon. Soc. Annu. Meet.* 63 2277–2281. 10.1177/1071181319631411

[B37] Ertl M. Hildebrandt M. Ourina K. Leicht G. Mulert C. (2013). Emotion regulation by cognitive reappraisal - the role of frontal theta oscillations. *NeuroImage* 81 412–421. 10.1016/j.neuroimage.2013.05.044 23689018

[B38] Felnhofer A. Kothgassner O. D. Schmidt M. Heinzle A. K. Beutl L. Hlavacs H. (2015). Is virtual reality emotionally arousing? Investigating five emotion inducing virtual park scenarios. *Int. J. Hum. Comput. Stud.* 82 48–56. 10.1016/j.ijhcs.2015.05.004

[B39] Freeman J. Avons S. E. Meddis R. Pearson D. E. Ijsselsteijn W. (2000). Using behavioral realism to estimate presence: A study of the utility of postural responses to motion stimuli. *Pres. Teleoperat. Virtual Environ.* 9 149–164. 10.1162/105474600566691

[B40] Friese U. Köster M. Hassler U. Martens U. Trujillo-Barreto N. Gruber T. (2013). Successful memory encoding is associated with increased cross-frequency coupling between frontal theta and posterior gamma oscillations in human scalp-recorded EEG. *Neuroimage* 66 642–647. 23142278 10.1016/j.neuroimage.2012.11.002

[B41] Fulvio J. Rokers B. (2018). Sensitivity to sensory cues predicts motion sickness in virtual reality. *J. Vis.* 18 1066–1066.

[B42] Gellhorn E. (1970). The emotions and the ergotropic and trophotropic systems - part I. The physiological control of the emotions. *Psychol. Forsch.* 34 48–66. 10.1007/BF00422862 4929035

[B43] Gold C. Fachner J. Erkkilä J. (2013). Validity and reliability of electroencephalographic frontal alpha asymmetry and frontal midline theta as biomarkers for depression. *Scand. J. Psychol.* 54 118–126. 10.1111/sjop.12022 23278257

[B44] Gorini A. Griez E. Petrova A. Riva G. (2010). Assessment of the emotional responses produced by exposure to real food, virtual food and photographs of food in patients affected by eating disorders. *Ann. Gen. Psychiatry* 9:30. 10.1186/1744-859X-9-30 20602749 PMC2914081

[B45] Greenberg D. L. Rubin D. C. (2003). The neuropsychology of autobiographical memory. *Cortex* 39 687–728. 10.1016/S0010-9452(08)70860-8 14584549

[B46] Gromer D. Madeira O. Gast P. Nehfischer M. Jost M. Müller M. (2018). Height simulation in a virtual reality cave system: Validity of fear responses and effects of an immersion manipulation. *Front. Hum. Neurosci.* 12:372. 10.3389/fnhum.2018.00372 30319376 PMC6167601

[B47] Gromer D. Reinke M. Christner I. Pauli P. (2019). Causal interactive links between presence and fear in virtual reality height exposure. *Front. Psychol.* 10:141. 10.3389/fpsyg.2019.00141 30761054 PMC6363698

[B48] Gruber T. Tsivilis D. Giabbiconi C.-M. Müller M. M. (2008). Induced electroencephalogram oscillations during source memory: Familiarity is reflected in the gamma band, recollection in the theta band. *J. Cogn. Neurosci.* 20 1043–1053. 18211247 10.1162/jocn.2008.20068

[B49] Grunwald M. Weiss T. Mueller S. Rall L. (2014). EEG changes caused by spontaneous facial self-touch may represent emotion regulating processes and working memory maintenance. *Brain Res.* 1557 111–126. 24530432 10.1016/j.brainres.2014.02.002

[B50] Harman J. Brown R. Johnson D. (2017). “Improved memory elicitation in virtual reality: New experimental results and insights,” in *IFIP conference on human-computer interaction*, eds Bernhaupt R. Dalvi G. Joshi A. Balkrishan D. O’Neill J. Winckler M. (Cham: Springer), 128–146. 10.1007/978-3-319-67684-5_9

[B51] Hertweck S. Weber D. Alwanni H. Unruh F. Fischbach M. Latoschik M. E. (2019). “Brain activity in virtual reality: Assessing signal quality of high-resolution EEG while using head-mounted displays,” in *Proceedings of the 2019 26th IEEE conference on virtual reality and 3D user interfaces, VR*, (Osaka), 970–971. 10.1109/VR.2019.8798369

[B52] Higuera-Trujillo J. L. López-Tarruella Maldonado J. Llinares Millán C. (2017). Psychological and physiological human responses to simulated and real environments: A comparison between photographs, 360^°^ panoramas, and virtual reality. *Appl. Ergon.* 65 398–409. 10.1016/j.apergo.2017.05.006 28601190

[B53] Hodges L. F. Kooper R. Meyer T. C. Rothbaum B. O. Opdyke D. de Graaff J. J. (1995). Virtual environments for treating the fear of heights. *Computer* 28 27–33. 10.1109/2.391038

[B54] Hofmann S. M. Klotzsche F. Mariola A. Nikulin V. V. Villringer A. Gaebler M. (2019). “Decoding subjective emotional arousal during a naturalistic VR experience from EEG using LSTMs,” in *Proceedings of the 2018 IEEE international conference on artificial intelligence and virtual reality, AIVR 2018*, (Taichung), 128–131. 10.1109/AIVR.2018.00026

[B55] Horvat M. Dobrinic M. Novosel M. Jercic P. (2018). “Assessing emotional responses induced in virtual reality using a consumer EEG headset: A preliminary report,” in *Proceedings of the 2018 41st international convention on information and communication technology, electronics and microelectronics, MIPRO*, (Opatija), 1006–1010. 10.23919/MIPRO.2018.8400184

[B56] IJsselsteijn W. de Ridder H. Freeman J. Avons S. (2000). Presence: Concept, determinants and measurement. *Proc. SPIE Int. Soc. Opt. Eng.* 3959:520. 10.1117/12.387188

[B57] Jensen O. Tesche C. D. (2002). Frontal theta activity in humans increases with memory load in a working memory task. *Eur. J. Neurosci.* 15 1395–1399. 10.1046/j.1460-9568.2002.01975.x 11994134

[B58] Kisker J. Gruber T. Schöne B. (2020). Virtual reality experiences promote autobiographical retrieval mechanisms: Electrophysiological correlates of laboratory and virtual experiences. *Psychol. Res.* 10.1007/s00426-020-01417-x 32930880 PMC8440245

[B59] Kisker J. Gruber T. Schöne B. (2021a). Behavioral realism and lifelike psychophysiological responses in virtual reality by the example of a height exposure. *Psychol. Res.* 85 68–81. 31520144 10.1007/s00426-019-01244-9

[B60] Kisker J. Gruber T. Schöne B. (2021b). Experiences in virtual reality entail different processes of retrieval as opposed to conventional laboratory settings: A study on human memory. *Curr. Psychol.* 40 3190–3197.

[B61] Kisker J. Lange L. Flinkenflügel K. Kaup M. Labersweiler N. Tetenborg F. (2021c). Authentic fear responses in virtual reality: A mobile EEG study on affective, behavioral and electrophysiological correlates of fear. *Front. Virt Real.* 2:106. 10.3389/frvir.2021.716318

[B62] Klimesch W. (1999). EEG alpha and theta oscillations reflect cognitive and memory performance: A review and analysis. *Brain Res. Rev.* 29 169–195. 10.1016/S0165-0173(98)00056-3 10209231

[B63] Klimesch W. (2012). Alpha-band oscillations, attention, and controlled access to stored information. *Trends Cogn. Sci.* 16 606–617. 10.1016/j.tics.2012.10.007 23141428 PMC3507158

[B64] Klotzsche F. Mariola A. Hofmann S. Nikulin V. V. Villringer A. Gaebler M. (2018). “Using EEG to decode subjective levels of emotional arousal during an immersive VR roller coaster ride,” in *Proceedings of the 2018 25th IEEE conference on virtual reality and 3D user interfaces, VR*, (Tuebingen), 605–606. 10.1109/VR.2018.8446275

[B65] Knyazev G. G. Savostyanov A. N. Levin E. A. (2004). Alpha oscillations as a correlate of trait anxiety. *Int. J. Psychophysiol.* 53 147–160. 10.1016/j.ijpsycho.2004.03.001 15210292

[B66] Kothgassner O. D. Felnhofer A. (2020). Does virtual reality help to cut the Gordian knot between ecological validity and experimental control? *Ann. Int. Commun. Assoc.* 44 210–218. 10.1080/23808985.2020.1792790

[B67] Krijn M. Emmelkamp P. M. G. Biemond R. de Wilde de Ligny C. Schuemie M. J. Van Der Mast C. A. P. G. (2004). Treatment of acrophobia in virtual reality: The role of immersion and presence. *Behav. Res. Ther.* 42 229–239. 10.1016/S0005-7967(03)00139-6 14975783

[B68] Krohne H. W. Egloff B. Kohlmann C. W. Tausch A. (1996). (PDF) Untersuchungen mit einer deutschen Version der “positive and negative affect schedule” (PANAS). *Diagnostica* 42 139–156.

[B69] Krokos E. Plaisant C. Varshney A. (2019). Virtual memory palaces: Immersion aids recall. *Virt. Real.* 23 1–15. 10.1007/s10055-018-0346-3

[B70] Lakens D. (2017). Equivalence tests: A practical primer for t tests, correlations, and meta-analyses. *Soc. Psychol. Personal. Sci.* 8 355–362. 28736600 10.1177/1948550617697177 PMC5502906

[B71] Lange L. Osinsky R. (2021). Aiming at ecological validity—Midfrontal theta oscillations in a toy gun shooting task. *Eur. J. Neurosci.* 54 8214–8224. 10.1111/ejn.14977 32954574

[B72] Laux L. Glanzmann P. Schaffner P. Spielberger C. (1981). *State-trait-angstinventar [German version of State-Trait-Anxiety Inventory].* Göttingen: Hogrefe.

[B73] Lee S. M. Jang K. I. Chae J. H. (2017). Electroencephalographic correlates of suicidal ideation in the theta-band. *Clin. EEG Neurosci.* 48 316–321. 10.1177/1550059417692083 28201930

[B74] Liebherr M. Corcoran A. W. Alday P. M. Coussens S. Bellan V. Howlett C. (2021). EEG and behavioral correlates of attentional processing while walking and navigating naturalistic environments. *Sci. Rep.* 11:22325. 10.1038/s41598-021-01772-8 34785702 PMC8595363

[B75] Lin J. H. T. (2017). Fear in virtual reality (VR): Fear elements, coping reactions, immediate and next-day fright responses toward a survival horror zombie virtual reality game. *Comput. Hum. Behav.* 72 350–361. 10.1016/j.chb.2017.02.057

[B76] Lucarotti C. Oddo C. M. Vitiello N. Carrozza M. C. (2013). Synthetic and bio-artificial tactile sensing: A review. *Sensors (Switzerland)* 13 1435–1466. 10.3390/s130201435 23348032 PMC3649411

[B77] Luft C. D. B. Bhattacharya J. (2015). Aroused with heart: Modulation of heartbeat evoked potential by arousal induction and its oscillatory correlates. *Sci. Rep.* 5:15717. 10.1038/srep15717 26503014 PMC4621540

[B78] MacArthur C. Grinberg A. Harley D. Hancock M. (2021). “You’re making me sick: A systematic review of how virtual reality research considers gender & cybersickness,” in *Proceedings of the 2021 CHI conference on human factors in computing systems*, (New York, NY), 1–15.

[B79] McLean C. P. Anderson E. R. (2009). Brave men and timid women? A review of the gender differences in fear and anxiety. *Clin. Psychol. Rev.* 29, 496–505. 10.1016/j.cpr.2009.05.003 19541399

[B80] Moran T. P. Bernat E. M. Aviyente S. Schroder H. S. Moser J. S. (2014). Sending mixed signals: Worry is associated with enhanced initial error processing but reduced call for subsequent cognitive control. *Soc. Cogn. Affect. Neurosci.* 10 1548–1556. 10.1093/scan/nsv046 25925270 PMC4631152

[B81] Murphy D. Skarbez R. (2022). What do we mean when we say “presence”? *Pres. Virt. Augment Real.* 29 171–190.

[B82] Nilsson N. C. Nordahl R. Serafin S. (2016). Immersion revisited: A review of existing definitions of immersion and their relation to different theories of presence. *Hum. Technol.* 12 108–134. 10.17011/ht/urn.201611174652

[B83] Nolan H. Whelan R. Reilly R. B. (2010). FASTER: Fully automated statistical thresholding for EEG artifact rejection. *J. Neurosci. Methods* 192 152–162. 10.1016/j.jneumeth.2010.07.015 20654646

[B84] Oing T. Prescott J. (2018). Implementations of virtual reality for anxiety-related disorders: Systematic review. *J. Med. Intern. Res.* 6:e10965. 10.2196/10965 30404770 PMC6249506

[B85] Opriş D. Pintea S. García-Palacios A. Botella C. Szamosközi Ş David D. (2012). Virtual reality exposure therapy in anxiety disorders: A quantitative meta-analysis. *Depress. Anxiety* 29 85–93. 10.1002/da.20910 22065564

[B86] Packheiser J. Berretz G. Rook N. Bahr C. Schockenhoff L. Güntürkün O. (2021). Investigating real-life emotions in romantic couples: A mobile EEG study. *Sci. Rep.* 11:1142. 10.1038/s41598-020-80590-w PMC7806608 33441947

[B87] Packheiser J. Schmitz J. Pan Y. El Basbasse Y. Friedrich P. Güntürkün O. (2020). Using mobile EEG to investigate alpha and beta asymmetries during hand and foot use. *Front. Neurosci.* 14:109. 10.3389/fnins.2020.00109 32116536 PMC7033815

[B88] Parsons T. D. (2015). Virtual reality for enhanced ecological validity and experimental control in the clinical, affective and social neurosciences. *Front. Hum. Neurosci.* 9:660. 10.3389/fnhum.2015.00660 26696869 PMC4675850

[B89] Perez C. C. (2019). *Invisible women: Exposing data bias in a world designed for men.* London: Vintage Books. 10.3399/bjgp20X709745 PMC7194768 32354824

[B90] Peterson S. M. Furuichi E. Ferris D. P. (2018). Effects of virtual reality high heights exposure during beam-walking on physiological stress and cognitive loading. *PLoS One* 13:e0200306. 10.1371/journal.pone.0200306 29979750 PMC6034883

[B91] Piccione J. Collett J. De Foe A. (2019). Virtual skills training: The role of presence and agency. *Heliyon* 5:e02583. 10.1016/j.heliyon.2019.e02583 31840112 PMC6893068

[B92] Putman P. Verkuil B. Arias-Garcia E. Pantazi I. Van Schie C. (2014). EEG theta/beta ratio as a potential biomarker for attentional control and resilience against deleterious effects of stress on attention. *Cogn. Affect. Behav. Neurosci.* 14 782–791. 10.3758/s13415-013-0238-7 24379166

[B93] Renoult L. Davidson P. S. R. Palombo D. J. Moscovitch M. Levine B. (2012). Personal semantics: At the crossroads of semantic and episodic memory. *Trends Cogn. Sci.* 16 550–558. 10.1016/j.tics.2012.09.003 23040159

[B94] Reyes del Paso G. A. Langewitz W. Mulder L. J. M. van Roon A. Duschek S. (2013). The utility of low frequency heart rate variability as an index of sympathetic cardiac tone: A review with emphasis on a reanalysis of previous studies. *Psychophysiology* 50 477–487. 10.1111/psyp.12027 23445494

[B95] Riva G. (2006). “Virtual reality,” in *Wiley encyclopedia of biomedical engineering*, ed. Akay M. (New York, NY: John Wiley & Sons), 1–10. 10.1007/978-3-319-98390-5_34-1

[B96] Riva G. Davide F. Ijsselsteijn W. (2003). *Being there: Concepts, effects and measurement of user presence in synthetic environments.* Amsterdam: IOS Press, 110–118.

[B97] Riva G. Mantovani F. Capideville C. S. Preziosa A. Morganti F. Villani D. (2007). Affective interactions using virtual reality: The link between presence and emotions. *Cyberpsychol. Behav.* 10 45–56. 10.1089/cpb.2006.9993 17305448

[B98] Rothbaum B. O. Hodges L. F. Kooper R. Opdyke D. Williford J. S. North M. (1995). Virtual reality graded exposure in the treatment of acrophobia: A case report. *Behav. Ther.* 26 547–554. 10.1016/S0005-7894(05)80100-5 7694917

[B99] Sadaghiani S. Scheeringa R. Lehongre K. Morillon B. Giraud A. L. Kleinschmidt A. (2010). Intrinsic connectivity networks, alpha oscillations, and tonic alertness: A simultaneous electroencephalography/functional magnetic resonance imaging study. *J. Neurosci.* 30 10243–10250. 10.1523/JNEUROSCI.1004-10.2010 20668207 PMC6633365

[B100] Salahuddin L. Cho J. Jeong M. G. Kim D. (2007). “Ultra short term analysis of heart rate variability for monitoring mental stress in mobile settings,” in *Proceedings of the 2007 29th annual international conference of the ieee engineering in medicine and biology society*, (Piscataway, NJ: IEEE), 4656–4659. 10.1109/IEMBS.2007.4353378 18003044

[B101] Sauseng P. Klimesch W. Doppelmayr M. Hanslmayr S. Schabus M. Gruber W. R. (2004). Theta coupling in the human electroencephalogram during a working memory task. *Neurosci. Lett.* 354 123–126. 10.1016/j.neulet.2003.10.002 14698454

[B102] Schipke A. G. (1999). Effect of respiration rate on short-term heart rate variability. *J. Clin. Basic Cardiol.* 2 92–95.

[B103] Schlink B. R. Peterson S. M. Hairston W. D. König P. Kerick S. E. Ferris D. P. (2017). Independent component analysis and source localization on mobile EEG data can identify increased levels of acute stress. *Front. Hum. Neurosci.* 11:310. 10.3389/fnhum.2017.00310 28670269 PMC5472660

[B104] Scholz S. Schneider S. L. Rose M. (2017). Differential effects of ongoing EEG beta and theta power on memory formation. *PLoS One* 12:e0171913. 10.1371/journal.pone.0171913 28192459 PMC5305097

[B105] Schöne B. Kisker J. Sylvester R. S. Radtke E. L. Gruber T. (2021). Library for universal virtual reality experiments (luVRe): A standardized immersive 3D/360^°^ picture and video database for VR based research. *Curr. Psychol.* 1–19. 10.1007/s12144-021-01841-1

[B106] Schöne B. Schomberg J. Gruber T. Quirin M. (2016). Event-related frontal alpha asymmetries: Electrophysiological correlates of approach motivation. *Exp. Brain Res.* 234 559–567. 10.1007/s00221-015-4483-6 26537961

[B107] Schöne B. Sylvester R. S. Radtke E. L. Gruber T. (2020). Sustained inattentional blindness in virtual reality and under conventional laboratory conditions. *Virt. Real.* 10.1007/s10055-020-00450-w

[B108] Schöne B. Wessels M. Gruber T. (2019). Experiences in virtual reality: A window to autobiographical memory. *Curr. Psychol.* 38 715–719. 10.1007/s12144-017-9648-y

[B109] Schubert T. Friedmann F. Regenbrecht H. (2001). The experience of presence: Factor analytic insights. *Pres. Teleoperat. Virt. Environ.* 10 266–281. 10.1162/105474601300343603

[B110] Schubring D. Schupp H. T. (2021). Emotion and brain oscillations: High arousal is associated with decreases in alpha- and lower beta-band power. *Cereb. Cortex* 31 1597–1608. 10.1093/cercor/bhaa312 33136146

[B111] Schubring D. Kraus M. Stolz C. Weiler N. Keim D. A. Schupp H. (2020). Virtual reality potentiates emotion and task effects of alpha/beta brain oscillations. *Brain Sci.* 10:537. 10.3390/brainsci10080537 32784990 PMC7465872

[B112] Serino S. Repetto C. (2018). New trends in episodic memory assessment: Immersive 360^°^ ecological videos. *Front. Psychol.* 9:1878. 10.3389/fpsyg.2018.01878 30333780 PMC6176050

[B113] Shafer D. M. (2021). The effects of interaction fidelity on game experience in virtual reality. *Psychol. Popular Media* 10:457.

[B114] Shafer D. M. Carbonara C. P. Korpi M. F. (2017). Modern virtual reality technology: Cybersickness, sense of presence, and gender. *Media Psychol. Rev.* 11:1.

[B115] Shaffer F. Ginsberg J. P. (2017). An overview of heart rate variability metrics and norms. *Front. Public Health* 5:258. 10.3389/fpubh.2017.00258 29034226 PMC5624990

[B116] Sheridan T. B. (1992). Musings on telepresence and virtual presence. *Pres. Teleoperat. Virt. Environ.* 1 120–126. 10.1162/pres.1992.1.1.120

[B117] Shiota M. N. Neufeld S. L. Yeung W. H. Moser S. E. Perea E. F. (2011). Feeling good: Autonomic nervous system responding in five positive emotions. *Emotion* 11 1368–1378. 10.1037/a0024278 22142210

[B118] Simeonov P. I. Hsiao H. Dotson B. W. Ammons D. E. (2005). Height effects in real and virtual environments. *Hum. Fact.* 47 430–438. 10.1518/0018720054679506 16170948

[B119] Singh H. Bauer M. Chowanski W. Sui Y. Atkinson D. Baurley S. (2014). The brain’s response to pleasant touch: An EEG investigation of tactile caressing. *Front. Hum. Neurosci.* 8:893. 10.3389/fnhum.2014.00893 25426047 PMC4226147

[B120] Slater M. (2009). Place illusion and plausibility can lead to realistic behaviour in immersive virtual environments. *Philos. Trans. R. Soc. B Biol. Sci.* 364 3549–3557. 10.1098/rstb.2009.0138 19884149 PMC2781884

[B121] Slater M. (2018). Immersion and the illusion of presence in virtual reality. *Br. J. Psychol.* 109 431–433. 29781508 10.1111/bjop.12305

[B122] Slater M. Sanchez-Vives M. V. (2016). Enhancing our lives with immersive virtual reality. *Front. Robot. AI* 3:74. 10.3389/frobt.2016.00074

[B123] Slater M. Gonzalez-Liencres C. Haggard P. Vinkers C. Gregory-Clarke R. Jelley S. (2020). The ethics of realism in virtual and augmented reality. *Front. Virt. Real.* 1:1. 10.3389/frvir.2020.00001

[B124] Slater M. Usoh M. Steed A. (1994). Depth of presence in virtual environments. *Pres. Teleoperat. Virt. Environ.* 3 130–144. 10.1162/pres.1994.3.2.130

[B125] Smith S. A. (2019). Virtual reality in episodic memory research: A review. *Psychon. Bull. Rev.* 26 1213–1237. 10.3758/s13423-019-01605-w 31037605

[B126] Stoffregen T. A. Bardy B. G. Merhi O. A. Oullier O. (2004). Postural responses to two technologies for generating optical flow. *Presence* 13 601–615.

[B127] Stoffregen T. A. Bardy B. G. Smart L. J. Pagulayan R. J. (2003). “On the nature and evaluation of fidelity in virtual environments,” in *Virtual and adaptive environments: Applications, implications, and human performance issues*, eds Hettinger L. J. Haas M. W. (Hillsdale, NJ: Lawrence Erlbaum Associates Publishers), 111–128.

[B128] Suetsugi M. Mizuki Y. Ushijima I. Kobayashi T. Tsuchiya K. Aoki T. (2000). Appearance of frontal midline theta activity in patients with generalized anxiety disorder. *Neuropsychobiology* 41 108–112. 10.1159/000026641 10644932

[B129] Taelman J. Vandeput S. Spaepen A. Van Huffel S. (2008). Influence of mental stress on heart rate and heart rate variability. *IFMBE Proc.* 22 1366–1369. 10.1007/978-3-540-89208-3_324

[B130] Usoh M. Catena E. Arman S. Slater M. (2000). Using presence questionnaires in reality. *Pres. Teleoperat. Virt. Environ.* 9 497–503. 10.1162/105474600566989

[B131] Uusberg A. Thiruchselvam R. Gross J. J. (2014). Using distraction to regulate emotion: Insights from EEG theta dynamics. *Int. J. Psychophysiol.* 91 254–260. 10.1016/j.ijpsycho.2014.01.006 24440597

[B132] Visser A. Büchel D. Lehmann T. Baumeister J. (2022). Continuous table tennis is associated with processing in frontal brain areas: An EEG approach. *Exp. Brain Res.* 240 1899–1909. 10.1007/s00221-022-06366-y 35467129 PMC9142473

[B133] Welch R. B. Blackmon T. T. Liu A. Mellers B. A. Welch R. B. Blackmon T. T. (1996). The effects of pictorial realism, delay of visual feedback, and observer interactivity on the subjective sense of presence. *Pres. Teleoperat. Visual Environ.* 5 263–273. 10.1162/pres.1996.5.3.263

[B134] Wilson M. L. Kinsela A. J. (2017). “Absence of gender differences in actual induced hmd motion sickness vs. pretrial susceptibility ratings,” in *Proceedings of the human factors and ergonomics society annual meeting*, Vol. 61 (Los Angeles, CA: SAGE Publications), 1313–1316.

[B135] Wolf E. Schüler T. Morisse K. (2020). “Impact of virtual embodiment on the perception of virtual heights,” in *Augmented reality and virtual reality*, eds Jung T. tom Dieck M. C. Rauschnabel P. A. (Cham: Springer), 197–211. 10.1007/978-3-030-37869-1_17

[B136] Zuckerman M. (1996). Item revisions in the sensation seeking scale form V (SSS-V). *Pers. Individ. Differ.* 20:515. 10.1016/0191-8869(95)00195-6

