# Supplementary material for: The reality of virtual reality
Source: Front Psychol. 2023 Feb 15;14:1093014. doi: 10.3389/fpsyg.2023.1093014 (PMC9975753; doi:10.3389/fpsyg.2023.1093014)
Supplement: Supplementary file 1 [file Table_1.pdf]

Supplementary Material

1 Supplementary Data

Supplementary video abstract: https://youtu.be/fPIrIajpfiA

2 Supplementary Figures and Tables

Supplementary Table S2. Test statistics for Kruskal-Wallis test as the non-parametric equivalent to the ANOVA, regarding differences between the three groups for the subjective measures (* p <.05, **p<.01, ***p<.001).

		Kruskal-Wallis test parameters		
		H	*df*	*p*
PANAS	positive affect, t_0	3.42	2	.181
	negative affect, t_0	1.08	2	.583
	positive affect, t_1	29.79	2	<.001***
	negative affect, t_1	3.11	2	.211
	positive affect, t_2	24.44	2	<.001***
	negative affect, t_2	5.68	2	.059
	Change in positive affect, t_1	25.22	2	<.001***
	Change in positive affect, t_2	25.72	2	<.001***
	Change in negative affect, t_1	1.93	2	.381
	Change in negative affect, t_2	1.99	2	.369
STAI	trait	0.45	2	.799
AQ	fear of height	0.96	2	.619
	Avoidance of height	0.68	2	.713

		Kruskal-Wallis test parameters		
		H	*df*	*p*
IPQ	General Presence	53.19	2	<.001***
	Spatial Presence	42.43	2	<.001***
	Involvement	28.58	2	<.001***
	Realness	44.48	2	<.001***

Supplementary Table S3. Test statistic for post-hoc Mann-Whitney U-tests and effect size r regarding the subjective measures. Significant differences and effect sizes are marked accordingly (* p <.05, **p<.01, ***p<.001; effect size: a = small effect, b = medium effect, c = large effect). Internal consistency was acceptable to good for all scales (Cronbach's alpha > .67 for all scales; see supplementary material 1, table S7 for details), with exception of PANAS negative affect t2.

		Descriptives			*Mann-Whitney U*-test			
		n	*Md*	*SD*	*U*	*z*	*p*	*effect size r*
IPQ: General Presence	RL	25	3.00	0.41	32.5	-5.65	<.001***	.81[c]
	VR	24	1.00	1.47				
	RL	25	3.00	0.41	0.0	-6.30	<.001***	.89[c]
	PC	25	-1.00	1.49				
	VR	24	1.00	1.47	117.5	-3.76	<.001***	.54[c]
	PC	25	-1.00	1.49				
IPQ: Spatial Presence	RL	25	9.00	3.15	111.5	-3.65	<.001***	.53[c]
	VR	23	4.00	5.06				
	RL	25	9.00	3.15	8.0	-5.92	<.001***	.84[c]
	PC	25	-3.00	5.69				
	VR	23	4.00	5.06	97.5	-3.93	<.001***	.57[c]
	PC	25	-3.00	5.69				

		Descriptives			*Mann-Whitney U*-test			
		n	*Md*	*SD*	*U*	*z*	*p*	*effect size r*
IPQ: Involvement	RL	21	-5.00	2.37	32.5	-5.03	<.001***	.75[c]
	VR	24	4.00	4.68				
	RL	21	-5.00	2.37	241.5	-0.47	.64	.07[a]
	PC	25	-4.00	4.86				
	VR	24	4.00	4.68	92.5	-4.16	<.001***	.59[c]
	PC	25	-4.00	4.86				
IPQ: Realness	RL	21	6.00	1.79	24.5	-5.21	<.001***	.78[c]
	VR	24	0.00	4.02				
	RL	21	6.00	1.79	2.0	-5.78	<.001***	.85[c]
	PC	25	-5.00	3.54				
	VR	24	0.00	4.02	151.0	-2.99	.003**	.43[b]
	PC	25	-5.00	3.54				
PANAS: positive affect. t1	RL	23	39.00	6.34	103.0	-3.56	<.001***	.53[c]
	VR	23	34.00	5.52				
	RL	23	39.00	6.34	52.0	-4.87	<.001***	.70[c]
	PC	25	27.00	6.44				
	VR	23	34.00	5.52	142.0	-3.00	.003**	.43[b]
	PC	25	27.00	6.44				
PANAS: positive affect. t2	RL	25	39.00	7.18	183.0	-2.34	.019*	.33[b]
	VR	24	32.00	7.29				
	RL	25	39.00	7.18	70.5	-4.70	<.001***	.67[c]
	PC	25	27.00	6.95				
	VR	24	32.00	7.29	152.0	-2.97	.003**	.42[b]
	PC	25	27.00	6.95				

		Descriptives			*Mann-Whitney U*-test			
		n	*Md*	*SD*	*U*	*z*	*p*	*effect size r*
Change in positive affect t1	RL	22	6.00	4.25	106.0	-3.20	.001**	.48[b]
	VR	22	0.00	6.26				
	RL	22	6.00	4.25	45.5	-4.90	<.001***	.72[c]
	PC	25	-3.00	5.18				
	VR	22	0.00	6.26	191.5	-1.79	.074	.26[a]
	PC	25	-3.00	5.18				
Change in positive affect t2	RL	24	6.00	4.51	179.0	-2.07	.039*	.30[b]
	VR	23	2.00	8.72				
	RL	24	6.00	4.51	47.5	-5.05	<.001***	.72[c]
	PC	25	-2.00	5.38				
	VR	23	2.00	8.72	150.5	-2.83	.005**	.41[b]
	PC	25	-2.00	5.38				

Supplementary Table S4. Test statistic for post-hoc Mann-Whitney U-tests and effect size r regarding differences in band power (* p <.05, **p<.01; ***p<.001; effect size: a = small effect, b = medium effect, c = large effect).

			Descriptives			*Mann-Whitney U*-test			
			n	*Md*	*SD*	*U*	*z*	*p*	*effect size r*
alpha power	baseline	RL	25	0.02	0.42	217.0	-1.66	.097	.24[a]
		VR	24	-0.23	0.55				
		RL	25	0.02	0.42	150.0	-3.15	.002**	.45[b]
		PC	25	-0.44	0.50				
		VR	24	-0.23	0.55	229.0	-1.42	.156	.20[a]

			Descriptives			*Mann-Whitney U*-test			
			n	*Md*	*SD*	*U*	*z*	*p*	*effect size r*
		PC	25	-0.44	0.50				
	ascend	RL	25	-0.78	0.42	293.0	-.14	.889	.02[a]
		VR	24	-0.64	0.63				
		RL	25	-0.78	0.42	127.0	-3.60	<.001***	.51[c]
		PC	25	-1.19	0.38				
		VR	24	-0.64	0.63	104.0	-3.92	<.001***	.56[c]
		PC	25	-1.19	0.38				
	highest point	RL	25	-1.56	0.60	292.0	-.16	.873	.02[a]
		VR	24	-1.32	0.92				
		RL	25	-1.56	0.60	150.0	-3.15	.002**	.45[b]
		PC	25	-1.96	0.42				
		VR	24	-1.32	0.92	127.0	-3.46	<.001***	.49[b]
		PC	25	-1.96	0.42				
	descend	RL	25	-0.34	0.47	281.0	-.38	.704	.05[a]
		VR	24	-0.29	0.53				
		RL	25	-0.34	0.47	209.0	-2.01	.045*	.28[a]
		PC	25	-0.58	0.47				
		VR	24	-0.29	0.53	186.0	-2.28	.023*	.33[b]
		PC	25	-0.58	0.47				
theta power	baseline	RL	25	0.80	0.52	98.0	-4.04	<.001***	.58[c]
		VR	24	0.15	0.45				
		RL	25	0.80	0.52	46. 0	-5.17	<.001***	.73[c]
		PC	25	-0.07	0.40				
		VR	24	0.15	0.45	211.0	-1.78	.075	.25[a]

			Descriptives			*Mann-Whitney U*-test			
			n	*Md*	*SD*	*U*	*z*	*p*	*effect size r*
		PC	25	-0.07	0.40				
	ascend	RL	25	-0.07	0.41	280.0	-.40	.689	.06[a]
		VR	24	0.01	0.65				
		RL	25	-0.07	0.41	101.0	-4.10	<.001***	.58[c]
		PC	25	-0.61	0.38				
		VR	24	0.01	0.65	89.0	-4.22	<.001***	.60[c]
		PC	25	-0.61	0.38				
	highest point	RL	25	-0.89	0.52	250.0	-1.00	.317	.14[a]
		VR	24	-0.75	0.95				
		RL	25	-0.89	0.52	103.0	-4.06	<.001***	.57[c]
		PC	25	-1.40	0.36				
		VR	24	-0.75	0.95	119.0	-3.62	<.001***	.52[c]
		PC	25	-1.40	0.36				
	descend	RL	25	0.32	0.45	219.0	-1.62	.105	.23[a]
		VR	24	0.17	0.43				
		RL	25	0.32	0.45	149.0	-3.17	.002**	.45[b]
		PC	25	-0.09	0.36				
		VR	24	0.17	0.43	195.0	-2.1	.035*	.30[b]
		PC	25	-0.09	0.36				
beta power	baseline	RL	25	-0.47	0.47	181.0	-2.38	.017*	.34[b]
		VR	24	-0.82	0.58				
		RL	25	-0.47	0.47	126.0	-3.62	<.001***	.51[c]
		PC	25	-0.97	0.38				

		Descriptives			Mann-Whitney *U*-test			
		n	*Md*	*SD*	*U*	*z*	*p*	*effect size r*
	VR	24	-0.82	0.58	276.0	-0.48	.63	.07[a]
	PC	25	-0.97	0.38				
ascend	RL	25	-1.36	0.48	168.0	-2.64	.008**	.38[b]
	VR	24	-0.83	0.77				
	RL	25	-1.36	0.48	101.0	-4.10	<.001***	.58[c]
	PC	25	-1.80	0.29				
	VR	24	-0.83	0.77	33.0	-5.34	<.001***	.76[c]
	PC	25	-1.80	0.29				
highest point	RL	25	-2.20	0.61	192.0	-2.16	.031*	.31[b]
	VR	24	-1.55	1.03				
	RL	25	-2.20	0.61	103.0	-4.06	<.001***	.57[c]
	PC	25	-2.61	0.36				
	VR	24	-1.55	1.03	46.0	-5.08	<.001***	.73[c]
	PC	25	-2.61	0.36				
descend	RL	25	-0.93	0.46	197.0	-2.06	.039*	.29[a]
	VR	24	-0.56	0.58				
	RL	25	-0.93	0.46	149.0	-3.17	.002**	.45[b]
	PC	25	-1.24	0.34				
	VR	24	-0.56	0.58	68.0	-4.64	<.001***	.66[c]
	PC	25	-1.24	0.34				

Supplementary Table S5. Test statistics for post-hoc Mann-Whitney U-tests and effect size r regarding both HRV parameters per phase of the ride in the firefighters' basket. Positive values indicate an increase, whereas negative values indicate a decrease compared to baseline. Significant differences between groups are marked respectively (* p <.05, **p<.01, ***p<.001; effect size: a = medium effect, b = large effect).

		Descriptives			*Mann-Whitney U*-test			
	group	*n*	*Md*	*SD*	*U*	*z*	*p*	*effect size r*
SDRR baseline	RL	21	54.29	32.28	164.0	-1.63	.104	.25[a]
	VR	22	38.18	39.93				
	RL	21	54.29	32.28	109.0	-3.13	.001**	.46[a]
	PC	25	115.17	35.63				
	VR	22	38.18	39.93	101.0	-3.74	<.001***	.55[b]
	PC	25	115.17	35.63				
Change in SDRR ascend	RL	20	26.01	49.17	159.0	-1.33	.183	.20[a]
	VR	21	14.24	30.90				
	RL	20	26.01	49.17	78.0	-3.93	<.001***	.59[b]
	PC	25	-57.58	35.37				
	VR	21	14.24	30.90	97.0	-3.66	<.001***	.54[b]
	PC	25	-57.58	35.37				
Change in SDRR highest point	RL	19	42.07	38.75	151.0	-1.52	.129	.24[a]
	VR	22	64.24	37.23				
	RL	19	42.07	38.75	122.0	-2.74	.006**	.41[b]
	PC	25	-12.75	33.68				
	VR	22	64.24	37.23	77.0	-4.22	<.001***	.62[b]
	PC	25	-12.75	33.68				

		Descriptives			*Mann-Whitney U*-test			
	group	*n*	*Md*	*SD*	*U*	*z*	*p*	*effect size r*
Change in SDRR descend	RL	18	-1.31	37.20	180.0	-0.49	.625	.08[a]
	VR	22	3.15	32.48				
	RL	18	-1.31	37.20	97.0	-3.15	.002**	.48[a]
	PC	25	-58.71	30.88				
	VR	22	3.15	32.48	99.0	-3.75	<.001***	.55[b]
	PC	25	-58.71	30.88				
Change in rmSSD baseline	RL	21	34.07	22.81	181.0	-1.22	.224	.19[a]
	VR	22	22.68	52.21				
	RL	21	34.07	22.81	104.0	-3.50	<.001***	.52[b]
	PC	25	77.27	26.50				
	VR	22	22.68	52.21	113.0	-3.46	.001**	.51[b]
	PC	25	77.27	26.50				
Change in rmSSD ascend	RL	20	2.04	38.93	196.0	-0.37	.715	.06[a]
	VR	21	0.61	46.31				
	RL	20	2.04	38.93	92.0	-3.61	<.001***	.54[b]
	PC	25	-45.53	23.13				
	VR	21	0.61	46.31	92.0	-3.76	<.001***	.55[b]
	PC	25	-45.53	23.13				
Change in rmSSD highest point	RL	19	54.91	34.06	94.0	-3.01	.003**	.44[a]
	VR	22	19.24	57.03				
	RL	19	54.91	34.06	45.0	-4.56	<.001***	.69[b]
	PC	25	-30.77	23.29				
	VR	22	19.24	57.03	93.0	-3.88	<.001***	.57[b]
	PC	25	-30.77	23.29				

	group	*Descriptives* *n*	*Md*	*SD*	*Mann-Whitney U*-test *U*	*z*	*p*	*effect size r*
Change in rmSSD descend	RL	18	-3.91	25.34	177.0	-0.57	.57	.09[a]
	VR	22	-4.18	47.96				
	RL	18	-3.91	25.34	98.0	-3.13	.001**	.48[a]
	PC	25	-50.20	24.71				
	VR	22	-4.18	47.96	122.0	-3.26	.001**	.48[a]
	PC	25	-50.20	24.71				

Supplementary Table S6. Shapiro Wilk tests for normal distribution. All variables were tested for normal distribution regarding each group using Shapiro-Wilk test. In case that at least one subgroup per variable or at least one subscale or subvariable of a measure was not normally distributed (p < .10), a non-parametric test was used for analysis of that measure. Significant test values indicating the negotiation of normal distribution were highlighted in grey below. Descriptive statistics are given per variable and group below. RL = real-life, VR = Virtual Reality, PC = personal computer.

variable	condition	Shapiro-Wilk *W*	*df*	*p*
PANAS_T0_positive_affect	RL	.970	24	.663
	VR	.988	23	.992
	PC	.958	25	.383
PANAS_T0_negative_affect	RL	.575	24	>.001
	VR	.709	21	>.001
	PC	.821	25	.001
STAIT_Trait	RL	.863	25	.003

variable	condition	Shapiro-Wilk		
		W	*df*	*p*
	VR	.841	24	.001
	PC	.890	25	.011
AQ: Fear of height	RL	.916	19	.094
	VR	.898	24	.019
	PC	.919	25	.048
AQ: Avoidance	RL	.941	22	.208
	VR	.928	24	.089
	PC	.605	25	>.001
Sensation Seeking	RL	.981	25	.912
	VR	.981	22	.929
	PC	.967	24	.600
IPQ General Presence	RL	.493	25	>.001
	VR	.843	24	.002
	PC	.847	25	.002
IPQ Spatial Presence	RL	.950	25	.246
	VR	.941	23	.188
	PC	.971	25	.659
IPQ Involvement	RL	.865	21	.008

variable	condition	Shapiro-Wilk		
		W	*df*	*p*
	VR	.970	24	.667
	PC	.970	25	.634
IPQ Realness	RL	.843	21	.003
	VR	.962	24	.480
	PC	.951	25	.263
PANAS_T1_positive_affect	RL	.967	23	.609
	VR	.913	23	.047
	PC	.976	25	.801
PANAS_T1_negative_affect	RL	.733	24	>.001
	VR	.576	23	>.001
	PC	.761	25	>.001
PANAS_T2_positive_affect	RL	.946	25	.208
	VR	.963	24	.509
	PC	.919	25	.049
PANAS_T2_negative_affect	RL	.913	25	.035
	VR	.850	24	.002
	PC	.949	25	.235
Change_PA_T1	RL	.952	22	.352

variable	condition	Shapiro-Wilk *W*	*df*	*p*
	VR	.976	22	.837
	PC	.981	25	.904
Change_PA_T2	RL	.947	24	.231
	VR	.917	23	.058
	PC	.956	25	.344
Change_NA_T1	RL	.685	23	>.001
	VR	.675	20	>.001
	PC	.841	25	.001
Change_NA_T2	RL	.708	24	>.001
	VR	.914	21	.064
	PC	.876	25	.006
Duration: ascent	RL	.751	25	>.001
	VR	.876	24	.007
	PC	.877	25	.006
Duration: descent	RL	.965	24	.544
	VR	.939	24	.153
	PC	.916	25	.041
Duration: highest point	RL	.729	24	>.001

variable	condition	Shapiro-Wilk		
		W	*df*	*p*
	VR	.827	24	.001
	PC	.821	25	.001
Duration: total ride	RL	.964	25	.492
	VR	.885	24	.011
	PC	.861	25	.003
Alpha_Baseline	RL	.967	25	.582
	VR	.960	24	.432
	PC	.981	25	.907
Alpha_ascent	RL	.945	25	.193
	VR	.748	24	>.001
	PC	.945	25	.194
Alpha_high	RL	.873	25	.005
	VR	.665	24	>.001
	PC	.959	25	.391
Alpha_descend	RL	.970	25	.647
	VR	.879	24	.008
	PC	.959	25	.401
Beta_Baseline	RL	.974	25	.746

variable	condition	Shapiro-Wilk		
		W	*df*	*p*
	VR	.954	24	.329
	PC	.963	25	.468
Beta_ascent	RL	.982	25	.925
	VR	.864	24	.004
	PC	.988	25	.990
Beta_high	RL	.940	25	.149
	VR	.734	24	>.001
	PC	.993	25	.999
Beta_descend	RL	.958	25	.382
	VR	.894	24	.016
	PC	.975	25	.778
Theta_Baseline	RL	.950	25	.249
	VR	.979	24	.876
	PC	.981	25	.895
Theta_ascent	RL	.959	25	.397
	VR	.745	24	>.001
	PC	.955	25	.318
Theta_high	RL	.906	25	.025

variable	condition	Shapiro-Wilk		
		W	*df*	*p*
	VR	.661	24	>.001
	PC	.957	25	.357
Theta_descend	RL	.974	25	.751
	VR	.905	24	.028
	PC	.969	25	.632
HRV_ascent_SDRR	RL	.972	20	.803
	VR	.963	23	.521
	PC	.975	25	.761
HRV_ascent_RMSSD	RL	.700	20	>.001
	VR	.965	23	.580
	PC	.952	25	.275
HRV_high_SDRR	RL	.923	19	.128
	VR	.959	24	.418
	PC	.858	25	.003
HRV_high_RMSSD	RL	.945	19	.320
	VR	.655	24	>.001
	PC	.745	25	>.001
HRV_descent_SDRR	RL	.931	18	.201

variable	condition	Shapiro-Wilk		
		W	*df*	*p*
	VR	.970	24	.663
	PC	.927	25	.073
HRV_descent_RMSSD	RL	.851	18	.009
	VR	.894	24	.016
	PC	.978	25	.850
HRV_baseline_SDRR	RL	.964	21	.610
	VR	.701	22	>.001
	PC	.817	25	>.001
HRV_baseline_RMSSD	RL	.951	21	.349
	VR	.614	22	>.001
	PC	.877	25	.006

Supplementary Table S7. Descriptive statistics per variable and group.

	real-life (RL) condition					virtual reality (VR) condition					personal computer (PC) condition				
	n		*M*	*Md*	*Std.-dev.*	*n*		*M*	*Md*	*Std.-dev.*	*n*		*M*	*Md*	*Std.-dev.*
	valid	missing				valid	missing				valid	missing			
PANAS T1 positive affect	24	1	31.83	32.50	5.63	23	1	31.26	32.00	5.99	25	0	29.08	30.00	5.17
PANAS T1 negative affect	24	1	13.71	12.50	4.78	21	3	14.14	12.00	5.41	25	0	12.92	12.00	3.37
STAIT trait	25	0	34.92	35.00	8.50	24	0	36.46	35.00	9.40	25	0	35.80	34.00	6.39
AQ fear of height	19	6	21.53	21.00	11.48	24	0	18.75	17.50	15.03	25	0	19.76	16.00	13.78
AQ avoidance of height	22	3	3.86	4.00	2.62	24	0	3.38	3.00	2.68	25	0	4.24	3.00	6.01
Sensation Seeking	25	0	21.80	23.00	6.10	22	2	20.86	21.00	5.61	24	1	20.79	20.00	5.03
IPQ General Presence	25	0	2.80	3.00	0.41	24	0	0.79	1.00	1.47	25	0	-1.04	-1.00	1.49

	real-life (RL) condition					virtual reality (VR) condition					personal computer (PC) condition				
	n		*M*	*Md*	*Std.-dev.*	*n*		*M*	*Md*	*Std.-dev.*	*n*		*M*	*Md*	*Std.-dev.*
	valid	missing				valid	missing				valid	missing			
IPQ Spatial Presence	25	0	9.12	9.00	3.15	23	1	3.26	4.00	5.07	25	0	-4.24	-3.00	5.68
IPQ Involvement	21	4	-4.29	-5.00	2.37	24	0	3.67	4.00	4.68	25	0	-3.20	-4.00	4.86
IPQ Realness	21	4	7.00	6.00	1.79	24	0	-0.58	0.00	4.02	25	0	-4.00	-5.00	3.54
PANAS T2 positive affect	23	2	39.65	39.00	6.34	23	1	32.70	34.00	5.52	25	0	27.20	27.00	6.44
PANAS T2 negative affect	24	1	10.79	10.00	1.14	23	1	10.39	10.00	0.78	25	0	10.96	10.00	1.27
PANAS T3 positive affect	25	0	37.60	39.00	7.18	24	0	32.54	32.00	7.29	25	0	25.32	27.00	6.95
PANAS T3 negative affect	25	0	15.00	15.00	2.69	24	0	15.21	14.00	3.67	25	0	13.32	13.00	2.15
Change in positive affect day 1	22	3	6.82	6.00	4.25	22	2	1.32	0.00	6.26	25	0	-1.88	-3.00	5.17
Change in positive affect day 2	24	1	5.54	6.00	4.51	23	1	1.78	2.00	8.72	25	0	-3.76	-2.00	5.38

	real-life (RL) condition					virtual reality (VR) condition					personal computer (PC) condition				
	n		*M*	*Md*	*Std.-dev.*	*n*		*M*	*Md*	*Std.-dev.*	*n*		*M*	*Md*	*Std.-dev.*
	valid	missing				valid	missing				valid	missing			
Change in negative affect day 1	23	2	-2.91	-2.00	4.52	20	4	-3.55	-2.00	4.87	25	0	-1.96	-1.00	2.82
Change in negative affect day 2	24	1	1.25	2.00	5.04	21	3	1.43	2.00	3.88	25	0	0.40	1.00	3.57
Duration ascend	25	0	149.52	144.00	17.70	24	0	152.50	147.50	11.36	25	0	151.85	147.00	11.61
duration descend	24	1	225.17	226.00	18.00	24	0	220.17	220.00	19.47	25	0	219.72	218.00	21.80
duration highest point	24	1	63.46	63.00	3.24	24	0	63.50	63.00	2.50	25	0	63.44	63.00	2.47
duration total ride	25	0	436.44	433.00	32.88	24	0	436.17	435.00	13.31	25	0	435.01	435.00	17.10
Alpha baseline	25	0	0.01	-0.02	0.42	24	0	-0.23	-0.22	0.55	25	0	-0.44	-0.54	0.50
Alpha ascend	25	0	-0.78	-0.83	0.42	24	0	-0.64	-0.80	0.63	25	0	-1.19	-1.18	0.38
Alpha highest point	25	0	-1.56	-1.48	0.60	24	0	-1.32	-1.60	0.92	25	0	-1.96	-1.95	0.42
Alpha descend	25	0	-0.34	-0.29	0.47	24	0	-0.29	-0.36	0.53	25	0	-0.58	-0.68	0.47

	real-life (RL) condition					virtual reality (VR) condition					personal computer (PC) condition				
	n		*M*	*Md*	*Std.-dev.*	*n*		*M*	*Md*	*Std.-dev.*	*n*		*M*	*Md*	*Std.-dev.*
	valid	missing				valid	missing				valid	missing			
Beta baseline	25	0	-0.47	-0.53	0.47	24	0	-0.82	-0.96	0.58	25	0	-0.97	-0.98	0.38
Beta ascend	25	0	-1.36	-1.38	0.48	24	0	-0.83	-1.01	0.77	25	0	-1.80	-1.77	0.29
Beta highest point	25	0	-2.20	-2.15	0.61	24	0	-1.55	-2.05	1.03	25	0	-2.61	-2.58	0.36
Beta descend	25	0	-0.93	-0.92	0.46	24	0	-0.56	-0.71	0.58	25	0	-1.24	-1.22	0.34
Theta baseline	25	0	0.80	0.69	0.52	24	0	0.15	0.18	0.45	25	0	-0.07	-0.06	0.40
Theta ascend	25	0	-0.07	0.02	0.41	24	0	0.01	-0.18	0.65	25	0	-0.61	-0.64	0.38
Theta highest point	25	0	-0.89	-0.83	0.52	24	0	-0.75	-1.04	0.95	25	0	-1.40	-1.44	0.36
Theta descend	25	0	0.32	0.32	0.45	24	0	0.17	0.16	0.43	25	0	-0.09	-0.04	0.36
SDNN ascend	20	5	83.21	84.65	29.66	23	1	57.93	56.78	17.94	25	0	55.96	52.92	17.58
rmSSD ascend	20	5	42.68	33.69	33.63	23	1	32.13	31.48	15.70	25	0	32.57	30.03	15.96
SDNN highest point	19	6	103.77	94.64	23.24	24	0	108.09	110.82	17.40	25	0	102.72	99.46	18.17

	real-life (RL) condition					virtual reality (VR) condition					personal computer (PC) condition				
	n		*M*	*Md*	*Std.-dev.*	*n*		*M*	*Md*	*Std.-dev.*	*n*		*M*	*Md*	*Std.-dev.*
	valid	missing				valid	missing				valid	missing			
rmSSD highest point	19	6	82.92	79.92	25.00	24	0	53.14	48.55	25.61	25	0	49.62	48.06	16.34
SDNN descend	18	7	57.31	55.45	17.52	24	0	46.56	47.04	19.65	25	0	51.33	54.45	16.74
rmSSD descend	18	7	32.56	25.39	17.20	24	0	25.62	22.97	17.77	25	0	30.62	33.30	12.07
SDNN baseline	21	4	59.28	54.29	32.28	22	2	49.13	38.18	39.93	25	0	99.56	115.17	35.63
rmSSD baseline	21	4	37.94	34.07	22.81	22	2	40.50	22.68	52.21	25	0	68.10	77.27	26.50

Supplementary Table S8. Cronbach's alpha for all used questionnaires. *Internal consistency was determined for each subscale of the used questionnaires. The individual number of items and scale ranges are given as well.*

questionnaire	subscale	number of items	scale range	Cronbach's alpha
PANAS	T1: positive affect	10	10 - 50	.86
	T1: negative affect	10	10 - 50	.87
	T2: positive affect	10	10 - 50	.92
	T2: negative affect	10	10 - 50	.27
	T3: positive affect	10	10 - 50	.93
	T3: negative affect	10	10 - 50	.71
STAI	trait, no subscales	20	20 - 80	.87
AQ	fear of height	20	0 - 120	.87
	avoidance of height	20	0 - 40	.67
SSS-V	no subscales	40	0 - 40	.77
IPQ	General Presence	1	(-3) - 3	-
	Spatial Presence	5	(-15) - 15	.77
	Involvement	4	(-12) - 12	.67
	Realness	4	(-12) - 12	.82

Supplementary Table S9. Test statistics for Kruskal-Wallis testregarding differences in band power. *Test statistics for Kruskal-Wallis test as the non-parametric equivalent to the ANOVA, regarding differences between the three groups for each phase and frequency band (*p <.05, **p<.01, ***p<.001).*

		Kruskal-Wallis test parameters		
frequency band	phase	*H*	*df*	*p*
alpha (8-12Hz)	baseline	9.941	2	.007**
	ascend	19.056	2	<.001***
	highest point	14.702	2	.001**
	descend	6.202	2	.045*
beta (15-20 Hz)	baseline	12.717	2	.002**
	ascend	31.273	2	<.001***
	highest point	26.425	2	<.001***
	descend	22.375	2	<.001***
theta (4-7Hz)	baseline	31.064	2	<.001***
	ascend	23.360	2	<.001***
	highest point	20.558	2	<.001***
	descend	11.457	2	.003**

Supplementary Table S10. Test statistics for Kruskal-Wallis test regarding differences in heart rate variability. *Test statistics for Kruskal-Wallis test as the non-parametric equivalent to the ANOVA, regarding differences between the three groups regarding Changes in the HRV parameters SDRR and rmSSD for the separate phases of the ride in the firetruck's basket (*p <.05, **p<.01, ***p<.001).*

		Kruskal-Wallis test parameters		
	phase	*H*	*df*	*p*
SDNN	Baseline	18,636	2	<.001***
	ascend	20,806	2	<.001***
	highest point	19,224	2	<.001***
	descend	17,006	2	<.001***
rmSSD	Baseline	17,167	2	<.001***
	ascend	18,893	2	<.001***
	highest point	29,283	2	<.001***
	descend	14,391	2	.001**

2.1 Supplementary Figures

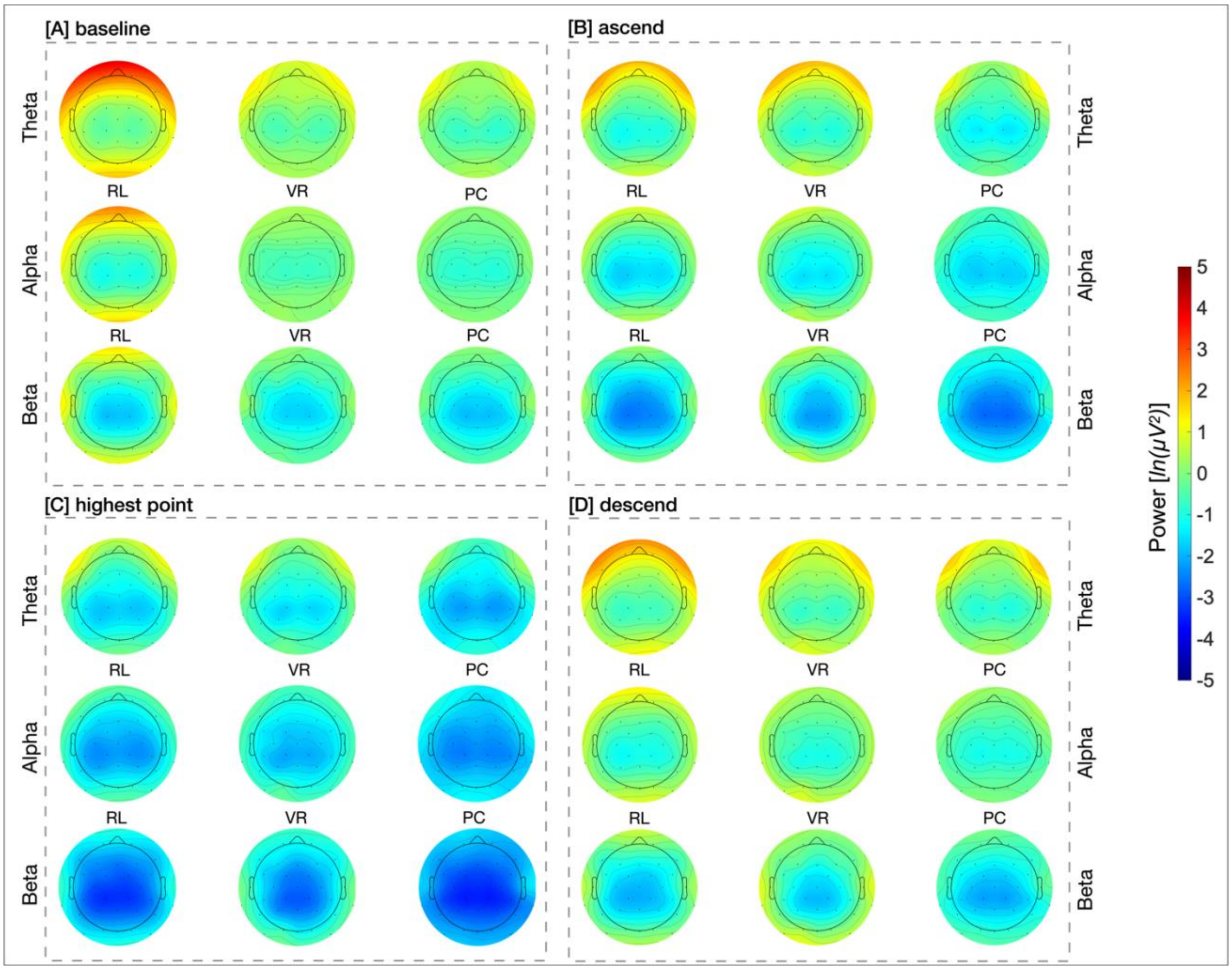

Supplementary Figure S11. Topographical distributions of the power in all examined frequency bands and phases of the ride per condition.
